# Inference of RNA Polymerase II Transcription Dynamics from Chromatin Immunoprecipitation Time Course Data

**DOI:** 10.1371/journal.pcbi.1003598

**Published:** 2014-05-15

**Authors:** Ciira wa Maina, Antti Honkela, Filomena Matarese, Korbinian Grote, Hendrik G. Stunnenberg, George Reid, Neil D. Lawrence, Magnus Rattray

**Affiliations:** 1 Department of Electrical and Electronic Engineering, Dedan Kimathi University of Technology, Nyeri, Kenya; 2 Helsinki Institute for Information Technology HIIT, Department of Computer Science, University of Helsinki, Helsinki, Finland; 3 Nijmegen Centre for Molecular Life Sciences, Radboud University Nijmegen, Nijmegen, The Netherlands; 4 Genomatix Software GmbH, Muenchen, Germany; 5 Institute for Molecular Biology, Mainz, Germany; 6 Department of Computer Science, University of Sheffield, Sheffield, United Kingdom; 7 Faculty of Life Sciences, University of Manchester, Manchester, United Kingdom; University of Illinois at Urbana-Champaign, United States of America

## Abstract

Gene transcription mediated by RNA polymerase II (pol-II) is a key step in gene expression. The dynamics of pol-II moving along the transcribed region influence the rate and timing of gene expression. In this work, we present a probabilistic model of transcription dynamics which is fitted to pol-II occupancy time course data measured using ChIP-Seq. The model can be used to estimate transcription speed and to infer the temporal pol-II activity profile at the gene promoter. Model parameters are estimated using either maximum likelihood estimation or via Bayesian inference using Markov chain Monte Carlo sampling. The Bayesian approach provides confidence intervals for parameter estimates and allows the use of priors that capture domain knowledge, e.g. the expected range of transcription speeds, based on previous experiments. The model describes the movement of pol-II down the gene body and can be used to identify the time of induction for transcriptionally engaged genes. By clustering the inferred promoter activity time profiles, we are able to determine which genes respond quickly to stimuli and group genes that share activity profiles and may therefore be co-regulated. We apply our methodology to biological data obtained using ChIP-seq to measure pol-II occupancy genome-wide when MCF-7 human breast cancer cells are treated with estradiol (E2). The transcription speeds we obtain agree with those obtained previously for smaller numbers of genes with the advantage that our approach can be applied genome-wide. We validate the biological significance of the pol-II promoter activity clusters by investigating cluster-specific transcription factor binding patterns and determining canonical pathway enrichment. We find that rapidly induced genes are enriched for both estrogen receptor alpha (ER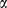) and FOXA1 binding in their proximal promoter regions.

## Author Summary

Cells express proteins in response to changes in their environment so as to maintain normal function. An initial step in the expression of proteins is transcription, which is mediated by RNA polymerase II (pol-II). To understand changes in transcription arising due to stimuli it is useful to model the dynamics of transcription. We present a probabilistic model of pol-II transcription dynamics that can be used to compute RNA transcription speed and infer the temporal pol-II activity at the gene promoter. The inferred promoter activity profile is used to determine genes that are responding in a coordinated manner to stimuli and are therefore potentially co-regulated. Model parameters are inferred using data from high-throughput sequencing assays, such as ChIP-Seq and GRO-Seq, and can therefore be applied genome-wide in an unbiased manner. We apply the method to pol-II ChIP-Seq time course data from breast cancer cells stimulated by estradiol in order to uncover the dynamics of early response genes in this system.

## Introduction

Transcription mediated by RNA polymerase II (pol-II) is an essential process in the expression of protein-coding genes in eukaryotes. Transcription is dependent upon a number of sequential and dynamic events, such as recruitment of pol-II to the transcriptional start site, activation of pol-II through phosphorylation of its C-terminal domain, elongation of the nascent transcript through the transcribed region and termination [1]. Each of these steps may be rate-limiting and can therefore affect the level of gene expression. In this paper, we describe a simple probabilistic model of transcription whose parameters can be inferred using time-series data such as pol-II ChIP-Seq data [2] or nascent transcript measurement by GRO-Seq that reports markers of transcriptional activity [3]. This model can be used to identify transcriptionally engaged genes, estimate their transcription rates and infer transcriptional activity adjacent to the promoter. The transcriptional dynamics of estrogen responsive genes in a breast cancer cell line were described by fitting this model to pol-II ChIP-seq time course datasets.

Chromatin immunoprecipitation, in conjunction with massively parallel sequencing (ChIP-seq) evaluates interactions between proteins and DNA, and, for example, can be used to monitor the presence of pol-II on DNA. Estimating the amount of pol-II associated with a transcribed gene provides a measure of transcriptional activity [2]. Sequential measurement of pol-II occupancy on genes released from transcriptional blockade, for example, in response to stimuli, reveal a wave of transcription moving through the body of the responding transcript.

A number of studies have attempted to determine the rate of transcription through modelling the dynamics of pol-II. Darzacq *et al.* fit a mechanistic model of pol-II transcription to nascent RNA data at a single locus and obtained a transcription speed of 4.3 kilobases per minute [4]. Wada *et al.* activated transcription of genes greater than 100 kbp in length and estimated the transcription speeds using a model that measures an intronic RNA signal through taking advantage of co-transcriptional splicing. They obtain an average transcription rate of 3.1 kbp min^−1^ [5]. Singh and Padget (2009) reversibly inhibit transcription to determine the transcription rate of 9 genes, all of which were greater than 100 kbp which had an average transcription rate of 3.79 kbp min^−1^ [6]. The data used in these studies have good temporal resolution (e.g. samples every 7.5 min in [5]) and reliably allow fitting of mathematical models or the direct measurement of transcription speed, however, only for a limited set of long genes. In contrast, high throughput data sets such as ChIP-Seq, can be used to uncover transcription dynamics genome-wide but typically have much lower temporal resolution, motivating the development of alternative modelling approaches that report genome-wide transcription rates.

One way around the low temporal resolution of typical high-throughput time course data is to employ a non-parametric model of the biological signals of interest. In many cases we expect these signals to vary continuously and smoothly in time, when averaged over a cell population, and a Gaussian process model provides a convenient non-parametric model in such cases [7]. Gaussian processes have recently found applications in a range of biological system models [8]–[11].

Here we present a Gaussian process model of transcription dynamics which can be fitted to genome-wide pol-II occupancy data measured using ChIP-Seq. The model describes the movement of pol-II through the gene body and combines a flexible model of promoter-proximal pol-II activity with a reliable estimate of transcription speed. By identifying genes which fit the model well, we provide a useful method to identify actively transcribed genes. The model does not assume a constant transcription speed and can therefore identify variable rates of transcription, for example due to transcriptional pausing. Model parameters are inferred using either maximum likelihood (ML) estimation or via Bayesian inference using Markov chain Monte Carlo (MCMC) sampling. The Bayesian approach provides confidence intervals for parameter estimates and can incoporate priors that capture domain knowledge, e.g. the expected range of transcription speeds, based on previous experiments.

We fit our model to a pol-II ChIP-Seq time course dataset from MCF7 breast cancer cells stimulated with estradiol. The model is used to identify the set of transcriptionally engaged genes and estimate their mean transcription rate and transcriptional activity near the promoter. By clustering promoter activity profiles, potential co-regulated groups of genes are identified, particularly those that respond rapidly to estrogen signalling. Subsequent characterisation of transcription factor (TF) binding sites in proximity to the promoters of genes within clusters provides a means of classifying groups of promoters that are responsive to the binding of specific combinations of TFs. Additionally, publically available ChIP-Seq datasets of TF profiles from the same system were used to identify cluster-specific patterns in TF-binding. The rates of transcription estimated by our model are consistent with the literature [4], [5] but with the advantage that our method allows the computation of transcription speeds genome-wide.

Our methodology has a number of advantages. We do not require data with high temporal resolution, making it feasible to model transcriptional dynamics genome-wide using ChIP-Seq or GRO-Seq time course data. We infer transcription rates for all genes in an unbiased manner and by using Bayesian parameter estimation we are able to associate our transcription rate estimates with confidence intervals. Our model is non-parametric and therefore does not make very strong assumptions about the temporal changes in transcriptional activity. Fitting the model genome-wide allows us to identify and filter out transcripts where pol-II does not travel down the gene body. This provides a principled method to identify responsive genes, in particular, early acting estrogen responsive genes in the specific application considered here. Since our model does not enforce a uniform transcription speed over the entire gene body, we can take into account phenomena such as pol-II pausing which would result in a non-uniform transcription speed. We also use this model to infer the promoter activity of transcriptionally engaged genes, to identify co-regulated gene modules downstream of estrogen signalling.

## Methods

**Figure 1:**
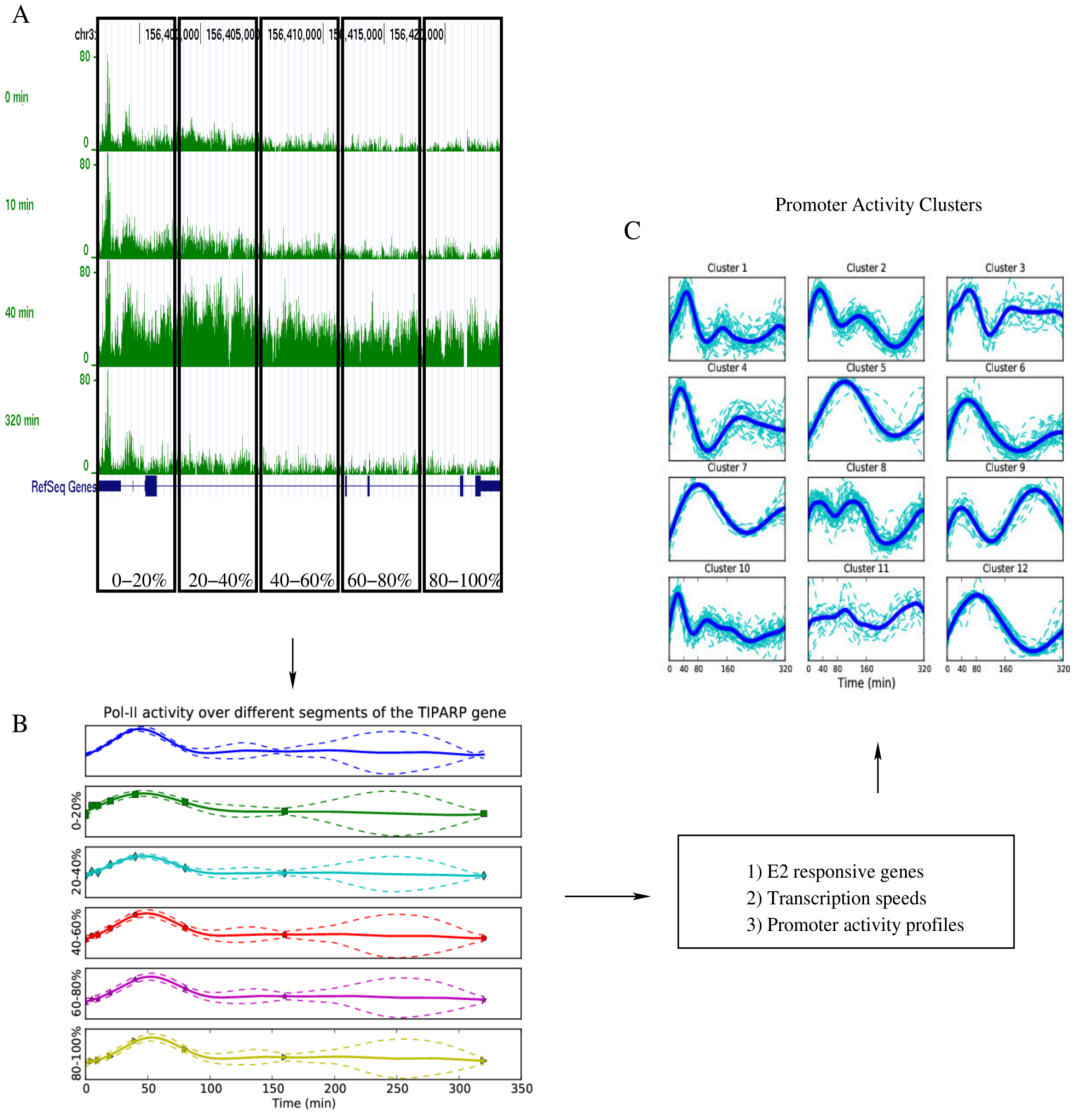
Description of the transcription dynamics modelling framework. Pol-II ChIP-seq data for the TIPARP gene shows a transcription wave moving down the gene. The transcription dynamics model captures this motion and allows us to estimate transcription speeds. In this case the gene is divided into 5 segments and we estimate the speed to be approximately 2 kilobases per minute. Panel A shows the raw ChIP-seq reads at different times between 0 and 320 min. The top part of panel B shows the inferred promoter activity profile. The next five parts of panel B show the inferred profiles for the five gene segments corresponding to 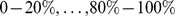 of the gene. By clustering these promoter activity profiles as shown in panel C, we are able to group genes into clusters that are likely to be co-regulated and in particular we identify the clusters that respond most rapidly to estrogen signalling.

Visualizing pol-II ChIP-seq reads mapped to transcriptional units at multiple time points following the addition of estradiol to MCF7 cells reveals the motion of pol-II through the gene body of estrogen responsive genes (see Figure 1). Computing the average pol-II occupancy over successive gene segments describes the motion of the transcription wave. Thereafter, fitting a model capable of smoothly interpolating between observed time points and by determining the time taken for pol-II to move from one gene segment to the next determines if pol-II is transcriptionally engaged on a given transcript and the speed at which it is moving through this transcriptional unit. We use a convolved Gaussian process to model the relationship between the pol-II signal at different regions of the gene and across time. Model parameters are determined using maximum likelihood (ML) or Bayesian inference via Markov chain Monte Carlo (MCMC) to determine genes of interest and moreover, in the case of MCMC, determine confidence intervals for our parameter estimates.

### Convolved Gaussian Process Model

A Gaussian process (GP) is a distribution over the space of functions. This distribution is completely specified by a mean function 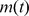 and a covariance function 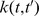. A function 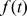 is said to be drawn from a Gaussian process 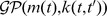 if 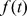 at any finite collection of points has a multivariate Gaussian distribution with mean vector and covariance matrix specified by 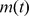 and 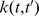, respectively. GPs provide a powerful framework for non-parametric regression [7]. If a function is assumed to be drawn from a GP with known mean and covariance function, we can infer the function value and associated uncertainty at unobserved locations given noise-corrupted observations. GPs have recently been applied in modelling biological systems, e.g. modelling protein concentrations as latent variables in differential equation models of transcriptional regulation [8], [9] and modelling spatial gene expression [11].

Here we introduce a novel application of GPs to modelling the spatio-temporal dynamics of pol-II occupancy during transcription. Convolved GPs allow the modelling of correlations between multiple coupled data sources. In our case these data sources are the pol-II occupancy over time collected at different locations along the transcribed region of a gene. Modelling the data as a convolved process borrows information from these different data sources in estimating the model parameters and inferring the underlying signal in the data. Also, we find that convolved GPs are necessary to account for changes in the shapes of signals observed at different regions of the gene. In linear systems theory, the output 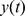 of a linear time-invariant system whose impulse response is 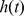 is given by the convolution of the input 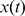 and 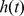, that is 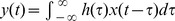. If different sets of observations are believed to be related, they can be modeled as the outputs of different linear systems in response to a single input. If this input is modeled as a GP, then it will form a joint GP together with all the outputs and data from one output stream will be useful in inferring the rest [12]–[20]. In our case, incorporating the data from multiple spatially separated regions of the genes allows us to infer an underlying function that links all these regions. This proves useful as a summary of the transcription dynamics of the gene and we show that it provides useful insights into potential coregulation.

#### Model description

In order to capture the movement of the transcription wave through transcriptional units, we divide each gene into 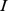 segments and compute time series of pol-II occupancy for each of the segments. Due to the low temporal resolution characteristic of high-throughput datasets, the time series between measurements must be inferred. To this end, we model the pol-II occupancy 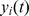 in each segment 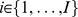 as the convolution of a latent process 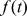 which is shared by all segments and a (possibly delayed) smoothing kernel 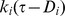 corrupted by an independent white Gaussian noise process 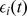 with zero mean and variance 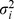 [15], [16]. That is 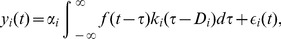
(1)where 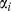 is a scale factor and 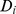 is the delay of each segment. The latent process 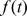 is modeled as a random function drawn from a GP with zero mean and a squared exponential covariance function (defined in Equation (4) below). The smoothing kernel is assumed to be Gaussian, that is
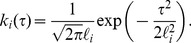
(2)


**Figure 2:**
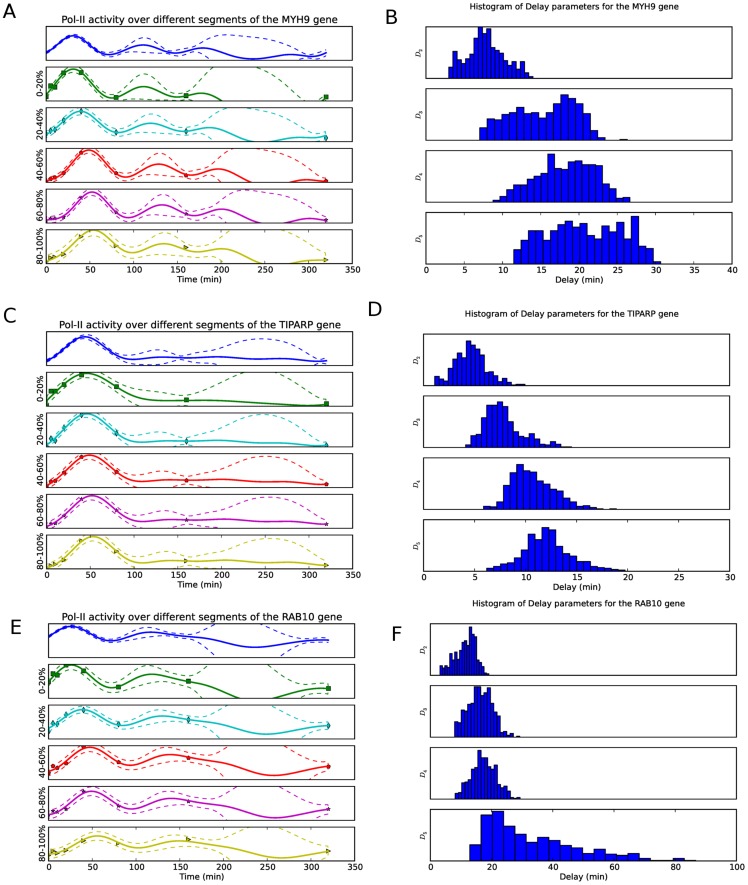
Inferred pol-II time profiles obtained for three of the top ten genes using ChIP-seq data. The panels on the left, (A,C,E) show the inferred distribution of the latent funtion 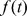 and the inferred profiles for the five gene segments corresponding to 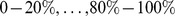 of the gene for the *MYH9*, *TIPARP* and *RAB10* genes respectively. We show the 95% confidence interval of the inferred profiles using dashed lines. The panels on the right (B,D,F) are the corresponding delay histograms.

The estimated delay 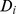 of each smoothing kernel models the amount of time it takes the ‘transcription wave’ to reach the corresponding gene segment. This is used to estimate the transcription speed. Biologically the latent function can be thought of as modeling activity at the promoter while the smoothing kernel accounts for ‘diffusion’ of the transcription wave. This diffusion phenomenon is observed when time series of pol-II occupancy over different sections of a gene are plotted, with the transcription wave seen to spread out (see Figure 2). This phenomenon may be due to an initially synchronized cell population becoming less synchronized over time, resulting in broadening of the pol-II occupancy distribution over time. The parameter 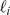 captures the amount of ‘spread’ observed at the 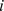 th segment. It also serves as a measure of the loss of synchrony between the cells of the population when the transcription wave is observed at the 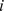 th segment.

Using equation (1), we can compute the covariance between the pol-II occupancy at various segments of the gene. We have
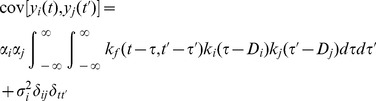
(3)where
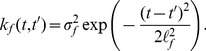
(4)


Equation (3) can be evaluated in closed form using the fact that the product of two Gaussians yields an un-normalized Gaussian [7]. Exploiting this fact we get
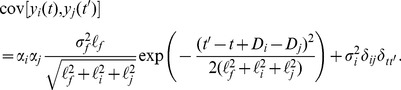
(5)


Similarly,
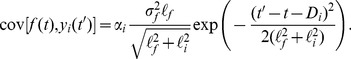
(6)


#### Parameter estimation and inference

Let 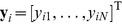 be a vector of observations of pol-II occupancy over the i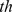 gene segment and let 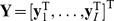 be a vector formed by concatenating all the observations for a single gene. 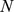 is the number of observation time points and 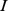 is the number of gene segments so for a single gene 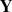 is a vector of length 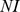. We have
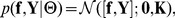
(7)where
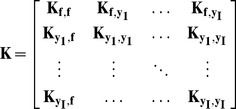
(8)and 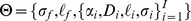 are the parameters of our model which will be fitted on a gene by gene basis. The elements of 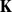 are computed using equations (4), (5), and (6). By marginalizing over the latent function 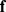, we obtain the marginal likelihood 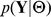. Maximum likelihood estimates of the parameters 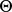 are readily obtained by maximizing the log marginal likelihood using gradient-based optimisation.

For a fully Bayesian approach, we take advantage of the fact that the parameters are positive and bounded. We transform the parameters using a logit transform and work with unconstrained variables. We place a Gaussian prior over the parameters in the transformed domain and draw samples from the posterior using the Hamiltonian Monte Carlo (HMC) algorithm [21] (A more detailed description of the priors is included in the supplementary material).

Code to implement the method is freely available as a Python package, PyPol-II, which can be downloaded from https://github.com/ciiram/PyPol_II.

#### Estimation of average transcription speed

When fitting the model, we fix 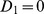 to ensure identifiability. The average transcription speed is computed by assuming that the value of 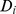 is an indicator of how long it takes the ‘transcription wave’ to reach the corresponding gene segment. That is, 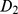 is the amount of time it takes to transcribe 20% of the gene, 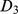 40% etc. To obtain confidence intervals on the delay estimates, MCMC was performed to get samples of the parameters.

To compute the average transcription speed we plot the position along the gene in base pairs (bp) versus the delay in minutes and compute a linear regression through the origin. The slope of the regression line gives us the transcriptional speed. Each sample of the parameters provides a set of delay estimates from which we obtain a speed estimate.

### Alternative Methods for Time Delay Inference

A key component of our method involves the estimation of delay between time series observed at different segments of the gene. The study of time delay between related time series has received attention from a number of researchers for a long time [22]. The application areas range from signal processing to astronomy [23]. The classic approach to time delay estimation involves computing the cross-correlation between the related time series and determining the value of delay for which this function is maximised. Consider two signals 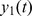 and 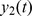 given by
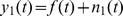


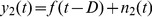
(9)where 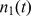 and 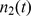 are uncorrelated noise processes. The cross-correlation function is given by 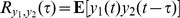 where 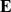 denotes the expectation operator. The value of 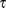 that maximises 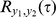 yields an estimate of the delay 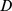. When the signals are sampled at 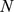 equally spaced time points 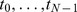 with spacing 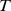 between samples, the discrete time equivalent of 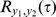 is readily estimated. Let 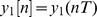, the discrete cross-correlation is estimated as
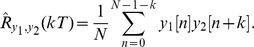



The delay is estimated by finding the value of 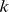 for which 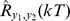 is maximised. The corresponding delay estimate is 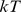. However, this approach does not work well when the time series are unevenly sampled as is the case in several astronomical and biological studies. A number of techniques have been developed to handle unevenly sampled time series including the discrete correlation function (DCF) [24], and the more recent kernel based approaches [25], [26]. The DCF is computed as follows, for all 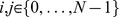 the time differences 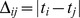 are binned into discrete bins of size 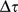. The DCF at 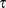 is given by [24], [25]

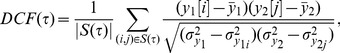
(10)where
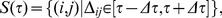
(11)and 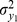 and 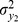 are the variances of the observation streams while 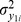 and 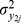 are observation error variances.

In the kernel based approach of [25], the underlying function 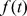 of equation (Equation 9) is modelled as the sum of a fixed number of kernels centered at the observation times. That is
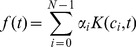
(12)where
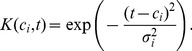
(13)


The value of 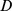 that minimises the estimation error is the delay estimate. Our implementation follows that presented in [25] where we assumed a fixed kernel width. This kernel width is determined by leave one out cross-validation.

### Benchmark Data

We used synthetic data and previously published experimental data to assess our novel method's performance. To generate the synthetic data, the underlying function 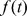 of equation (Equation 9) was given as a sum of Gaussian kernels. That is
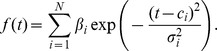



N was fixed at 20 and the observation interval 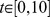. 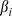, 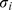 and 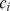 were generated at random with 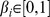, 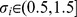 and 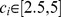. A random delay 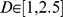 was used to generate the observations which were corrupted by additive Gaussian noise with 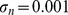. To determine the effect of number of observations on the quality of inference we compute the Median Normalised Square Error (MNSE) of the estimated delay 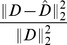 as a function of the number of observations for 50 random realisations of the the signals. We also investigated the effect of distorting the shape of the observed signals by introducing convolution. In real signals the restriction that the shape remains unchanged sometimes leads to poor results. The parameters of the smoothing kernel in equation (1) were generated at random with 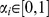 and 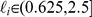.

To assess performance of our method on a well characterised real-world dataset we obtained a dataset from Singh and Padgett [6] where the delay in appearance of pre-mRNA signal at exon-intron junctions was used to compute estimates of transcription speed for 9 genes. To generate the data, transcription was reversibly inhibited *in vivo* using 5,6-dichlorobenzimidazole 1-beta-D-ribofuranoside (DRB) and the pre-mRNA measured after the inhibitor was removed. As verified by the authors, the kinetics of pol-II and pre-mRNA are similar hence we expect good performance on this dataset to indicate applicability of our method to pol-II ChIP-seq data.

### Pol-II ChIP-Seq Data

To demonstrate an application to pol-II ChIP-Seq data, we apply our model to investigate the transcriptional response to Estrogen Receptor signalling. ChIP-seq was used to measure pol-II occupancy genome-wide when MCF-7 breast cancer cells are treated with estradiol (E2). Cells were put in estradiol free media for three days. This is defined media devoid of phenol red (which is estrogenic) containing 2% charcoal stripped foetal calf serum. The charcoal absorbs estradiol but not other essential serum components, such as growth factors. This results in basal levels of transcription from E2 dependent genes. The cells are then incubated with E2 containing media, which results in the stimulation of estrogen responsive genes. The measurements were taken at logarithmically spaced time points 0, 5, 10, 20, …, 320 minutes after E2 stimulation.

Raw reads were mapped onto the human genome reference sequence (NCBI_build37) using the Genomatix Mining Station (software version 3.2.1). The mapping software on the Mining Station is an index based mapper that uses a shortest unique subword index generated from the reference sequence to identify possible read positions. A subsequent alignment step is then used to get the highest-scoring match(es) according to the parameters used. We used a minimum alignment quality threshold of 92% for mapping and trimmed 2 basepairs from the ends of the reads to account for deterioration in read quality at the 3′ end. The software generates separate output files for uniquely mapped reads and reads that have multiple matches with equal score. We only used the uniquely mapped reads. On average about 66% of all reads could be mapped uniquely. The data are available from the NCBI Gene Expression Omnibus under accession number GSE44800.

Time series of pol-II occupancy over various segments of genes were computed in reads per million (RPM) [27] using BEDtools [28], [29]. The genes were divided into 200 bp bins and the RPM computed for each bin. The occupancy in a particular gene segment was the mean RPM of the bins in that segment. Here, the gene is divided into five segments each representing 20% of the gene.

## Results

### Assessment on Benchmark Data

We first applied our methodology to synthetic data in order to compare its performance to other methods. We investigated the performance of five methods, namely cross-correlation (Corr), DCF, the kernel approach of [25] (Kern), a GP approach with no convolution (GP-NoConv), and the convolved GP approach developed in this paper (GP-Conv). Tables 1 and 2 show the MNSE for the different delay estimation methods as a function of the number of observations for synthetic data without convolution and with convolution respectively. Note that the kernel and DCF methods require an estimate of the noise variance and in this simulation study we provide the algorithms with the true value, but that would not be known in practice. We see that when no convolution is introduced, the kernel method performs well but is outperfomed by both GP methods. When convolution is introduced the kernel method appears to break down and as expected the GP-Conv outperforms the other techniques.

**Table 1:** MNSE as a function of the number of observations with no convolution.

Number of Observations	MNSE				
	Corr	DCF	Kern [25]	GP-NoConv	GP-Conv
6	36e-3	30e-3	4e-3	1.6e-3	2.2e-3
8	44e-3	48e-3	1.0e-3	0.16e-3	0.17e-3
10	11e-3	13e-3	1.2e-3	0.0076e-3	0.012e-3
12	19e-3	18e-3	1.2e-3	0.0018e-3	0.0014e-3

**Table 2:** MNSE as a function of the number of observations with convolution.

Number of Observations	MNSE				
	Corr	DCF	Kern [25]	GP-NoConv	GP-Conv
6	32e-3	37e-3	17000e-3	0.16e-3	0.053e-3
8	57e-3	61e-3	16000e-3	0.098e-3	0.0057e-3
10	11e-3	15e-3	17000e-3	0.018e-3	0.0021e-3
12	22e-3	31e-3	23000e-3	0.028e-3	0.011e-3

**Figure 3:**
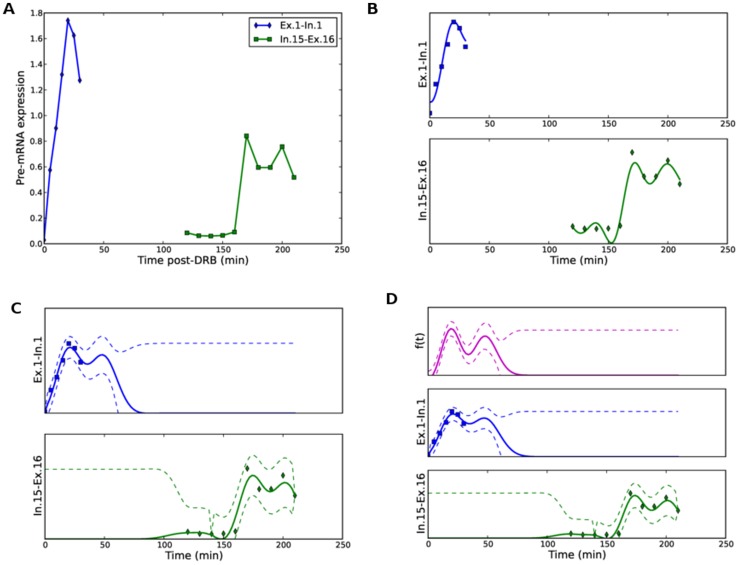
Pre-mRNA espression data. Pre-mRNA espression at exon-intron junctions for the *SLC9A9* gene (A). Fits for the *SLC9A9* gene using the kernel method (B) and the two GP methods: GP_NoConv (C) and GP_Conv (D). In the GP case we show the 95% confidence interval using dashed lines. In regions with no observations, the uncertainty is large.

We next applied the model to pre-mRNA data from Singh and Padgett [6] where the delay in appearance of pre-mRNA signal at exon-intron junctions was used to compute estimates of transcription speed for 9 genes. Figure 3(A) shows the pre-mRNA signal for the *SLC9A9* gene (the same data shown in Figure 4d of [6]). The delays read from these plots were used in [6] to determine transcription speeds. Figure 3 (B–D) shows the fit obtained using the kernel method, GP-NoConv and GP-Conv respectively. Table 3 shows the delays read off the plots as well as values obtained using the five delay estimation algorithms for different regions of the nine genes presented in [6]. In each row the delay estimate with the lowest normalised square error is highlighted. Table 4 shows the MNSE for the five delay estimation algorithms for all the genes. We see that the convolved GP method developed in this paper outperforms the other techniques. This method has the added advantage of inferring a latent function which links all the observations and which can be used for downstream analysis. Also, when analysis is genome-wide, reading delays off individual plots is not feasible and furthermore when the sampling intervals are irregularly spaced assigning delays manually would be error prone. These results serve to justify the use of the convolved GP method introduced in this paper.

**Figure 4:**
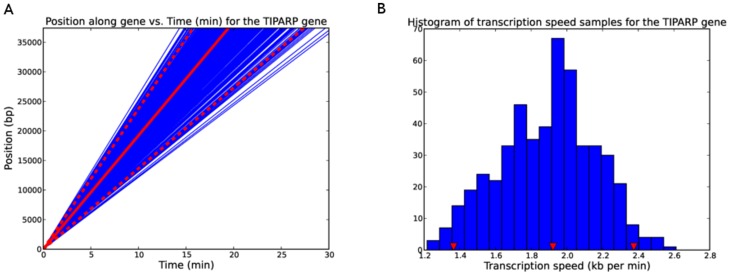
Computation of transcription speed from delay samples. Linear regression plots using the delay samples for the *TIPARP* gene (A) and the histogram of speed samples (B). The 95% confidence interval is indicated in (A) by the dashed red lines with the median represented by the solid red line. In (B) the 95% confidence interval is indicated by the red triangle markers (cf. Table 5).

**Table 3:** Transcription time estimates for different delay estimation algorithms using the pre-mRNA data from [6].

Gene	Region	Length (kb)	Delay (min)[6]	Corr	DCF	Kern [25]	GP	GP
							NoConv	Conv
Utrophin	Ex1-Ex2	111	30	15.0	10.8	3.1	46.9	**17.4**
Utrophin	Ex2-Ex50	174	40	-	49.2	125.5	49.5	**46.8**
Utrophin	Ex50-Ex51	101	25	-	10.8	67.3	**34.1**	13.8
Utrophin	Ex51-Ex74	173	40	-	238.3	214.3	9.9	**68.5**
Utrophin	Ex1-Ex74	561	140	-	135.6	128.6	**140.3**	146.4
ITPR1	Ex1-Ex5	133	40	45.0	45.5	**41.3**	49.2	43.2
ITPR1	Ex5-Ex40	105	25	**25.0**	24.8	23.0	17.4	24.0
ITPR1	Ex1-Ex40	238	65	70.0	69.8	96.4	**66.6**	67.2
EFNA5	Ex1-Ex2	243	70	65.0	65.4	146.9	69.8	**69.9**
BCL2	Ex2-Ex3	189	50	5.0	**54.9**	81.3	65.0	55.0
OPA1	Ex1-Ex29	104	25	20.0	**25.0**	14.9	27.0	26.8
IFT80	Ex1-Ex20	142	35	40	74.6	**35.2**	41.6	41.6
CTNNBL1	Ex1-Ex16	178	45	**45.0**	45.4	39.1	47.2	47.1
KIFAP3	Ex1-Ex20	153	45	**45.0**	45.4	39.1	46.7	46.7
SLC9A9	Ex1-Ex16	583	160	-	150.2	152.0	**153.6**	153.5

When sampling times are uneven, cross-correlation results are omitted. In each row the delay estimate with the lowest normalised square error is highlighted.

**Table 4:** MNSE for the 5 delay estimation algorithms for all the genes using pre-mRNA data.

	Corr	DCF	Kern [25]	GP-NoConv	GP-Conv
MNSE	0.115	1.787	1.974	0.090	**0.065**

### Application to Estrogen Response ChIP-Seq Data

We applied our method to a ChIP-Seq time-course dataset measuring pol-II occupancy genome-wide when MCF-7 cells are treated with estradiol (E2). For our initial experiment, we considered 3,064 genes which exhibit significant increase of pol-II occupancy between 0 and 40 minutes after E2 treatment. These genes were determined by counting the number of pol-II tags on the annotated genes in the RefSeq hg19 assembly at 0 and 40 minutes after E2 treatment and computing the 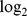 ratio of these counts. We keep those genes where this quantity is greater than one standard deviation above the mean. For these 3,064 genes, we filtered out genes less than 1000 bp in length and computed model fits using the ChIP-seq time series data for the remaining 2623 genes. The estimation of the parameters 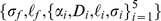 for a given gene was performed using maximum likelihood with 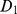 fixed at zero, 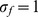 and the values 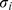 constrained to be equal. Intuitively, one would expect the values of delay 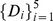 to be non-decreasing. We therefore keep only those genes where this natural ordering is preserved for further analysis. We also discard genes with 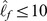 and 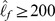 since these are generally seen to be poor fits. Small values of 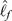 arise when the data is best modelled as a noise process while large values model constant profiles which are not interesting in our analysis. This left us with 383 genes which we consider a conservative set of genes where there is evidence of engaged transcription and where the model parameters can be confidently estimated. To rank these genes we compared the log marginal likelihood of the model fit to that obtained if we assume independence between the segments, which is equivalent to setting the off-diagonal blocks in equation (8) to the zero matrix.

Figure 2 (A–F) shows the inferred pol-II time profile and histogram of the samples of the delay parameters for three of the top 10 genes found to fit the model well. We note that a relatively small number of activated genes fit the model well. This is primarily because for shorter genes the pol-II occupancy quickly rises over the whole gene such that the temporal resolution of the data cannot capture the wave as it traverses the gene body. With a closer or more evenly spaced time course we would expect a good fit for a greater proportion of activated genes.

**Figure 5:**
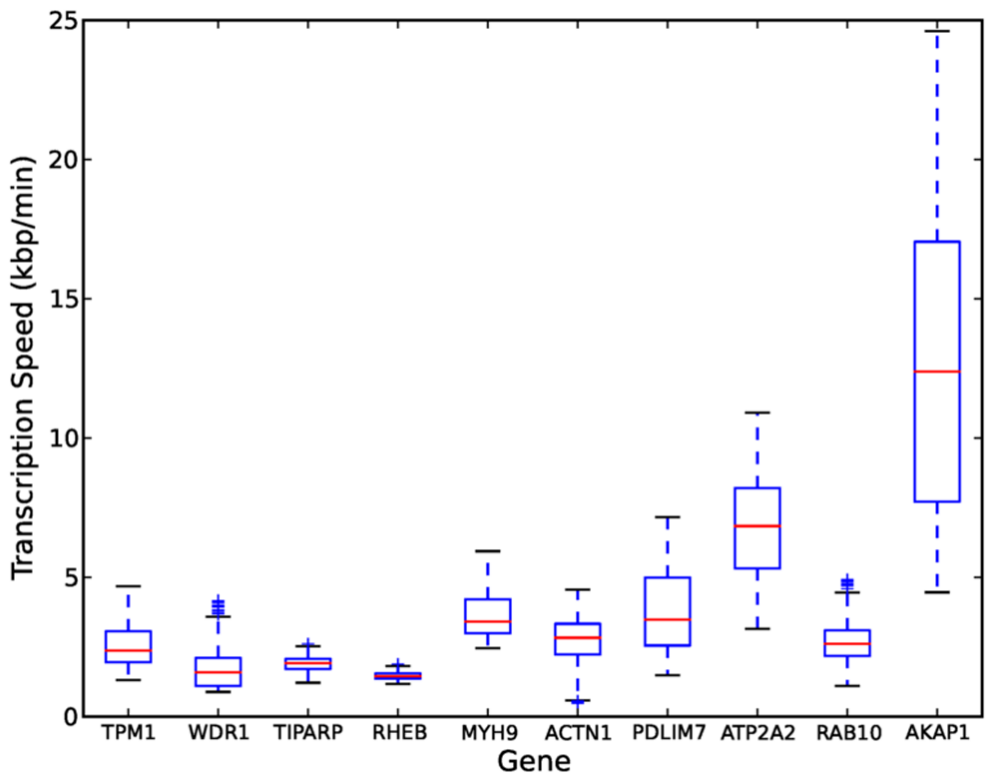
Box plot of speed estimates for the top ten genes found to fit the transcription model well. The box indicates the 50% confidence interval between the first and third quartiles. The red line indicates the median speed. The length of the whiskers is 1.5 times the interquartile range.

Figure 4 (A) shows the linear regression plots using the delay samples for the *TIPARP* gene. Figure 4 (B) shows the histogram of speed samples from which we can compute the confidence interval for the speed estimate. The 95% confidence interval is indicated in Figure 4 (B) by the red triangle markers (cf. Table 5). Table 5 shows the average transcription speeds for the top 10 genes computed using the samples of the delay parameters. Figure 5 shows a box plot of the average transcription speeds computed using the samples of the delay parameters for these genes.

**Table 5:** Average transcription speed in kilobases per minute for the top ten genes that fit the transcription model well.

Gene	Length (bp)	2.5%	50%	97.5%
TPM1	22196	1.6	2.4	4.1
WDR1	42611	1.0	1.6	3.5
TIPARP	32353	1.4	1.9	2.4
RHEB	53913	1.2	1.5	1.7
MYH9	106741	2.6	3.4	5.5
ACTN1	105244	0.6	2.8	4.2
PDLIM7	14208	1.7	3.5	6.4
ATP2A2	69866	3.6	6.8	10.2
RAB10	103595	1.4	2.6	4.4
AKAP1	36158	5.0	12.4	21.4

We use a Bayesian MCMC method for parameter estimation which provides the posterior distribution of the average transcription speed. We show the 2.5%, 50% and 97.5% percentiles of the posterior distribution.

The advantage of fitting each of the delay parameters independently instead of enforcing a linear relationship is that it allows us to take into account phenomena such as pol-II pausing and provides a means to filter genes where the values of estimated delay are not naturally ordered. Visual inspection of the inferred time series of the top ranked genes is consistent with a ‘transcription wave’ traversing the gene. The transcription wave is especially evident in the longer genes *MYH9* and *RAB10*. This motivates a closer look at long genes. Table 6 shows the average transcription speeds computed using the samples of the delay parameters for the 23 long genes found to fit the pol-II dynamics model well. Grouping these genes according to the magnitude of the median transcription speed allows us to compare our results to those presented previously. From Table 6 we see that 12 (52%) of these genes have average transcription speeds between 2 and 4 kb per minute, a range that includes speeds previously reported in the literature [5], [6].

**Table 6:** Average transcription speed in kilobases per minute for long genes between 100 and 300 kilobases long.

Gene	Length (bp)	2.5%	50%	97.5%
ACTN1	105244	0.6	2.8	4.2
ADCY1	148590	2.8	9.7	43.6
ARHGEF10L	158041	2.8	5.4	8.5
EPB41L1	120374	0.2	0.4	2.0
EPS15L1	110355	16.1	30.0	43.1
FARP1	102125	1.7	2.9	7.9
FLNB	163856	0.2	1.5	3.7
ITPK1	179005	0.3	2.9	6.8
JAK1	133282	0.6	2.2	4.2
JAK2	142939	0.6	2.4	5.3
KIAA0232	101441	0.9	2.3	4.0
KIF21A	150163	1.0	2.1	3.8
LARP1	104702	0.7	2.0	3.8
MYH9	106741	2.6	3.4	5.5
NCOR2	243050	6.5	10.9	20.5
NRIP1	103571	2.9	4.7	6.4
PKIB	116142	0.6	1.0	2.4
RAB10	103595	1.4	2.6	4.4
RAB31	154326	0.7	1.6	3.0
RASA3	150902	0.6	1.4	6.0
SHB	153316	0.5	3.1	5.0
WWC1	180244	1.9	3.6	5.6
ZNF644	106174	0.1	0.2	1.5

#### Clustering of promoter activity profiles

The inferred latent functions for each gene model the pol-II activity adjacent to the promoter. Clustering these profiles and examining the average profiles of each cluster allows us to visualise the general trends and also classify genes according to the immediacy and nature of the response. This provides an alternative to clustering based on mRNA abundance data (from microarray or RNA-Seq experiments) which is regulated both by mRNA production and degradation processes. The production of mRNA may be delayed relative to the actual activation of transcription at the promoter causing genes which are actually triggered at the same time to show different rates of mRNA production. Differences in degradation rate can also influence mRNA abundance profiles. It may therefore be difficult to distinguish early and delayed transcriptional regulation from mRNA abundance data.

To classify the profiles we sample the mean of the latent function (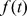 in equation 1) and use PUMA-CLUST [30] to cluster the genes. PUMA-CLUST has the advantage of taking into account the uncertainty of the latent function when clustering the profiles. This uncertainty is computed from the posterior covariance of 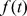.

**Figure 6:**
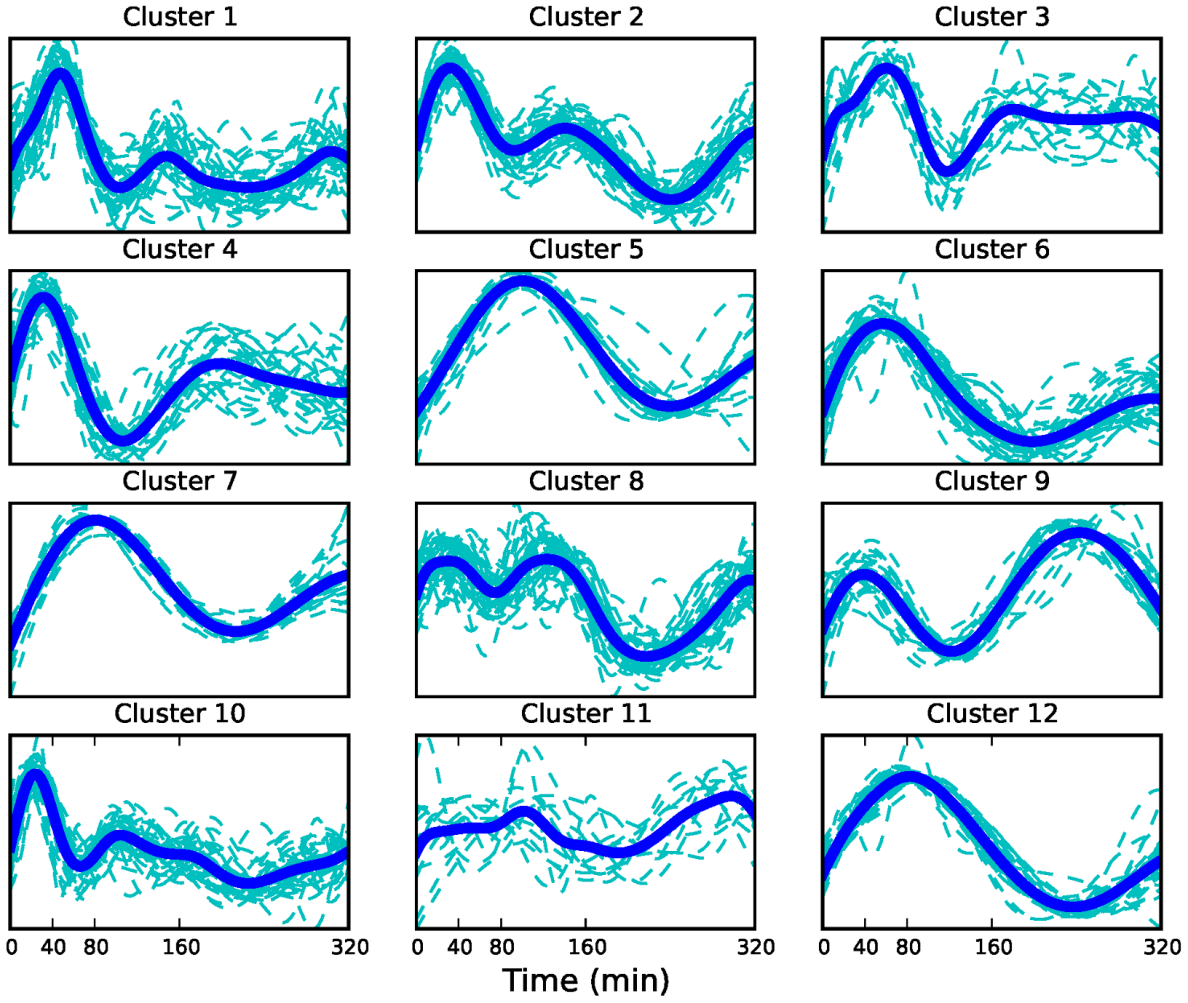
Clusters of promoter activity profiles. The mean profile in each cluster is shown by the bold line.

**Figure 7:**
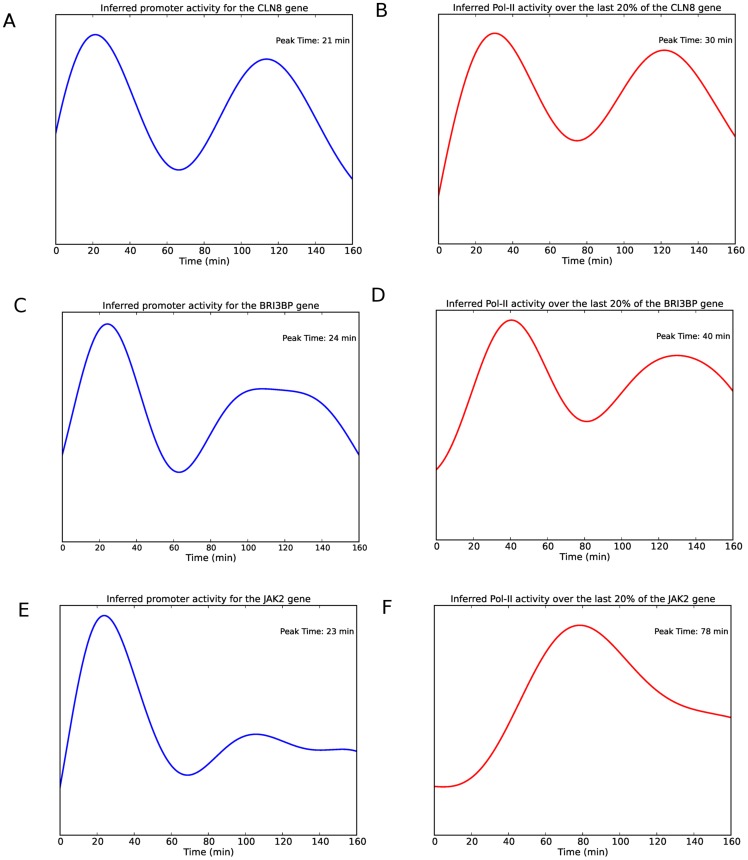
Influence of gene length on transcription time. Inferred promoter profiles and pol-II activity over the final 20% of the gene for three genes in cluster 10. The panels on the right (A,C,E) show the inferred promoter profiles while the panels on the left (B,D,F) show the corresponding pol-II activity over the final 20% of the gene.

**Figure 8:**
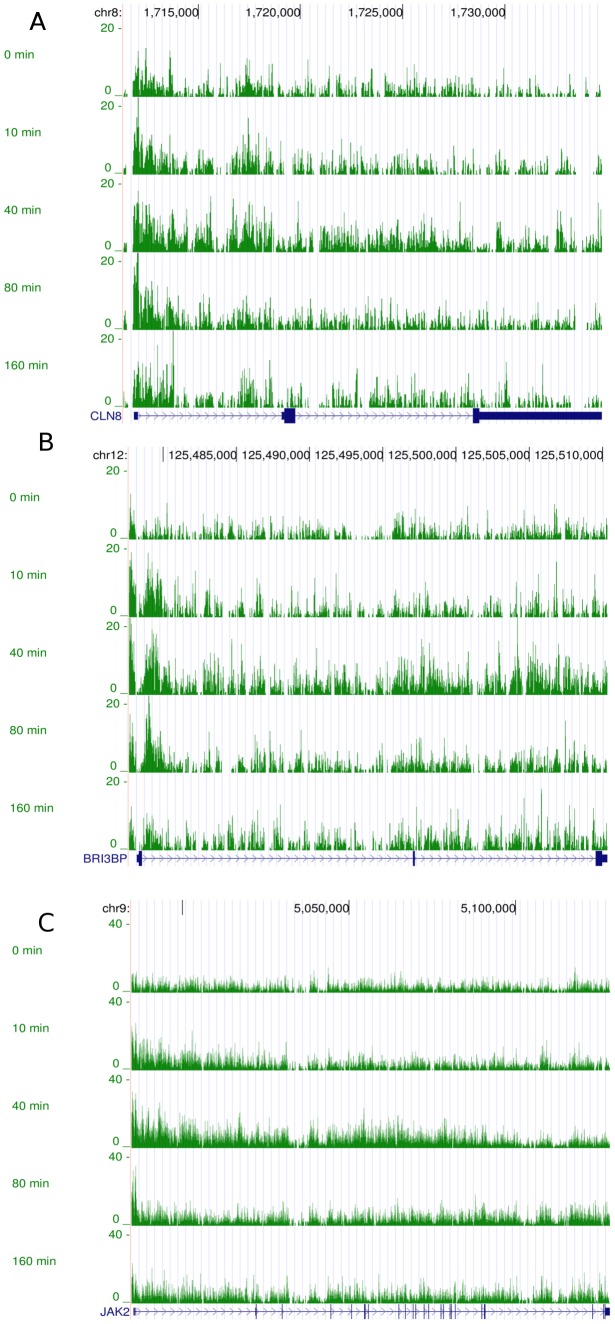
Raw ChIP-seq data. ChIP-seq reads for three genes in cluster 10: *CLN8* (A), *BRI3BP* (B) and *JAK2* (C).

The 383 genes found to fit the model well were grouped into 12 clusters (Figure 6) with the optimal number of clusters determined by the Bayesian Information Criterion. To determine the speed of the response in each cluster, we compute the peak time of the mean profile for each cluster (see Table 7). We used the Genomatix Pathway System (GePS) to look for enriched canonical pathways (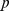-value 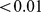) in each cluster (supplementary material, Table S4 in Text S1) and performed a Gene Ontology (GO) analysis of the clusters using the DAVID tool [31], [32] (supplementary material, Tables S5-S7 in Text S1) showing that clusters are enriched for a number of different GO categories. The GO analysis identified early peaking clusters such as 2, 4 and 10 as enriched for nucleotide binding proteins consistent with many early genes being involved in downstream transcriptional regulation. The clustering of the pair of genes *JAK1* and *JAK2* in cluster 10, which has a prominent early peak, suggests that the response of both genes to E2 is rapid and coordinated. Since these genes are known to act together in several biological pathways such as the IL-6 signaling pathway and the IFN gamma signaling pathway, their appearance in the same cluster suggests that the clustering is likely to reveal other biologically significant relationships. A closer look at the inferred pol-II promoter profiles of some examples in cluster 10, the earliest peaking cluster, and the corresponding inferred pol-II profiles over the last 20% of the genes reveals the possible influence of gene length on mRNA production and how clustering the inferred promoter profiles can account for this influence and uncover potential co-regulation. Figure 7 shows the inferred promoter profiles and the inferred pol-II profiles over the last 20% for three genes *CLN8*, *BRI3BP* and *JAK2* in cluster 10. Figure 8 shows the corresponding raw ChIP-seq reads. The lengths of the genes to the nearest kilobase are 23, 32 and 143 kb respectively. We see that despite the last segment profiles peaking at different times, the promoter profiles peak at approximately the same time. The difference in peak time over the final segment of the gene is most likely due to the length of the genes and accounts for the amount of time the pol-II takes to move down the gene. Such differences would mask potential co-regulation if we attempted to cluster genes based on their mRNA profiles.

**Table 7:** Peak time of the mean profile for each of the 12 clusters.

Cluster	Peak Time (min)
1	48
2	32
3	61
4	32
5	100
6	58
7	80
8	122
9	242
10	22
11	297
12	80

Clusters 1, 2, 4 and 10 have relatively early peaks.

In Hah *et al.* [3] GRO-seq was used to measure pol-II occupancy genome-wide when MCF-7 cells are treated with estradiol (E2) at four time points (0, 10, 40 and 160 min after E2 treatment). In addition, steady state levels of mRNA for 54 genes were measured using RT-qPCR at five time points (0, 10, 40, 160 and 320 min after E2 treatment). These data show a delay of between 1-3hr between peaks in the pol-II occupancy at the 5′ end of a gene and peaks in the mRNA steady state [3, Figure S4]. These data include the mRNA measurement for 20 genes whose corresponding GRO-seq data peak is at 40 minutes after E2 treatment. Six of these genes namely *CASP7, FHL2, GREB1, ITPK1, NRIP1, WWC1* are found to fit our pol-II model well with ChIP-seq data. Table 8 shows the peak time of the inferred promoter profile 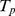, the peak time of the inferred pol-II profile over the last 20% of the gene 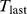, the GRO-seq peak time as well as the mRNA peak time. For the GRO-seq and mRNA peak times we show the peak times from Hah *et al.* [3, Figure S4] which are limited to the finite set of sampling times. We see that all mRNA peaks occur after 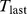. The large value of 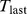 for *WWC1* which is a long gene 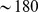 kb in length corresponds to a late peak in mRNA at 320 minutes. This shows that the parameters obtained by our model are biologically plausible. Based solely on the GRO-seq data these genes were grouped together in [3] since they show a peak at 40 min. However our modeling reveals a greater diversity in the nature of responses. In fact the six genes appear in three different early response promoter profile clusters (see Table 8).

**Table 8:** The peak time of the inferred promoter profile 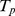, the peak time of the inferred pol-II profile over the last 20% of the gene 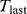, the GRO-seq peak time as well as the mRNA peak time (from [3, Figure S4]).

Gene	Cluster	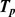	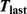	GRO-seq Peak	mRNA Peak
CASP7	1	36	47	40	160
FHL2	1	42	55	40	160
GREB1	2	30	46	40	320
ITPK1	2	36	64	40	160
NRIP1	10	22	40	40	160
WWC1	10	23	81	40	320

In the supplementary material, we compare the clustering obtained from the inferred promoter profiles to that obtained if the time series of the raw ChIP-seq reads are clustered and show that our model has the potential to uncover relationships which may be missed if we only consider the raw ChIP-seq reads.

#### Transcription factor binding

We investigated the TF peaks in a 40 kbp region around the gene transcription start site for all genes in each cluster using ChIP-seq data for a number of TFs measured under similar experimental conditions (i.e. MCF-7 breast cancer cells treated with E2) in the cistrome database (http://cistrome.org). In earlier work on the estrogen interactome, Fullwood *et al.* [33] suggest that most long range interactions between TF binding sites and gene enhancers are limited to a range of about 20 kb. We therefore investigate the region from −20 kb to 20 kb relative to the TSS (results for other regions around the TSS ranging from 1 to 100 kb are shown in the supplementary material (Tables S11–S14 in Text S1)). Table 9 shows the number of genes with TF binding peaks for each cluster for 7 TFs namely ER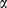 [2], FoxA1 [34], c-Fos [35], c-Jun [35], c-MYC [36], SRC-3 [37], TRIM24 [38]. We found that the TFs RAD21 [39], CTCF [39] and STAG1 [39] are ubiquitously bound and not useful in uncovering cluster-specific TF binding. We investigate the statistical significance of the proportions of genes in each cluster with TF peaks in a 40 kb neighborhood of the TSS by comparing the observed proportions to those we would expect in clusters of the same size drawn at random from the set of all genes. In Table 9 statistically significant (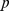-value 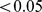) proportions are indicated in bold (larger than expected). For 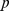-values less than 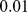, the associated 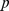-values are indicated in parentheses according to the following scale (***: 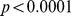,**: 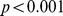,*: 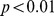).

**Table 9:** Analysis of transcription factor binding in 40

Cluster	TFs						
	ER 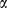	FOXA1	c-FOS	c-JUN	MYC	SRC-3	TRIM24
1 (37)	**27** (**)	**14**	**16** (*)	6	4	**25** (*)	27
2 (47)	**31** (*)	**19** (*)	**16**	7	**7**	**36** (***)	**38**
3 (18)	11	5	7	**5**	**6** (**)	11	12
4 (29)	**20** (*)	**11**	9	**7**	2	18	23
5 (27)	15	4	6	**8** (*)	**9** (***)	16	19
6 (40)	**27** (*)	8	12	7	4	**25**	31
7 (24)	10	6	5	**6**	3	13	19
8 (47)	**32** (*)	10	14	**14** (**)	**8**	**31** (*)	**40** (*)
9 (26)	**18**	7	**11** (*)	**11** (***)	3	12	**22**
10 (38)	**30** (***)	**14**	**15** (*)	2	1	**29** (**)	**32** (*)
11 (13)	5	2	**7** (*)	**4**	2	7	**13** (*)
12 (37)	19	8	12	**11** (**)	4	**23**	29

The number in parentheses in the first column is the cluster size. For each TF, we show the number of genes with peaks. Statistically significant proportions (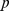-value 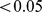) are indicated in bold (larger than expected). For 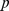-values less than 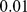, the associated 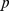-values are indicated in parentheses according to the following scale (***: 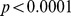,**: 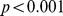,*: 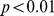).

Interestingly, clusters 1, 2, 4, and 10, which show an early peak in the mean promoter profile, are all enriched for ER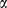 and FOXA1. These clusters, with the exception of cluster 4, were also found to be enriched for the ER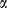 motif near the promoter. The enrichment of both ER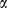 and FOXA1 in these clusters is in line with conclusions drawn in Hurtado *et al.* [40] where it was suggested FOXA1 mediates ER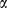 binding. We also investigated the overlap of the binding sites for ER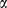 and FOXA1 both in the 151 genes belonging to these clusters and genome-wide using the peaks obtained from [2] (ER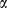) and [34] (FOXA1) and reported in the cistrome database. We investigated the 40 kb region −20 kbp to 20 kbp relative to the TSS. Table 10 shows the number of ER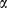 and FOXA1 peaks and the overlap (Two peaks are said to overlap if they have at least one base pair in common). We see that when we consider the rapid response genes in clusters 1, 2, 4, and 10 the percentage of overlap increases to 16% (35/220) whereas the overlap is 9% (956/11056) when we consider all genes. The significance associated with this elevated overlap is p = 0.004 given the null hypothesis of a random gene list of the same size (results for other regions around the TSS ranging from 1 to 100 kb are shown in the supplementary material (Tables S15 -S18 in Text S1)). Taken together, the results in Tables 9 and 10 identify genes that respond to E2, with clusters 1, 2, 4 and 10 most likely to contain the earliest estrogen responsive genes.

**Table 10:** Overlap of ER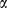 and FOXA1 binding in a 40 kb region around the TSS.

Genes	# of ER 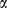 peaks	# of FOXA1 peaks	ER 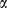 and FOXA1 overlap
Clusters 1, 2, 4, and 10 (151)	220 (112)	86 (44)	35 (0.004)
All genes ( 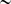 20,000)	11056	4626	956

The numbers in parentheses in the first column are the number of genes. In each TF peak column, we show the expected number of peaks in a set of random random genes of the same size in parentheses. In the overlap column the associated p-value is shown in parentheses.

## Discussion

In this work we have presented a methodology for modelling transcription dynamics and employed it to determine the transcriptional response of breast cancer cells to estradiol. To capture the movement of pol-II down the gene body, we model the observed pol-II occupancy time profiles over different gene segments as the delayed response of linear systems to the same input. The input is assumed to be drawn from a Gaussian process which models the pol-II activity adjacent to the gene promoter. Given observations from high-throughput data such as pol-II ChIP-Seq data, we are able to infer this input function and estimate the pol-II activity at the promoter. This allows us to differentiate transcriptionally engaged pol-II from pol-II paused at the promoter and yields good estimates of transcriptional activity.

In addition to estimating the transcriptional activity at the promoter, inferring the pol-II occupancy time profiles over different gene segments allows us to compute the transcription speed. We expect the delay parameters of different gene segments to be non-decreasing and this provides a natural way to determine genes that are being actively transcribed in response to E2.

Clustering the inferred promoter activity profiles allows us to investigate the nature of the response and group genes that are likely to be co-regulated. We found that the four clusters significantly enriched for both ER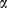 and FOXA1 binding within 40 kb according to public ChIP-Seq data were those that showed the earliest peak in pol-II activity at the promoter. ER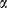 and FOXA1 ChIP peaks in the neighbourhood of these genes were also more likely to be overlapping than the average for ChIP-identified binding events of these TFs genome-wide. This observation provides some support for the previously proposed role of FOXA1 as a mediator of early transcriptional response in estrogen signalling. These results also show that our method can help regulatory network inference. The inferred promoter activity profiles pinpoint the times of transcriptional activation very accurately without confounding transcriptional delays. As genes with similar inferred promoter activity profiles are likely to have similar TF binding profiles, they are likely to be co-regulated as well. The promoter profiles should therefore lead to more accurate predictions of regulator-target relationships using time-course-based methods (e.g. [9]) than using expression time course data.

As well as modelling transcriptional speed and transcriptional activity profiles, the proposed modelling approach may have other useful applications. For example, recent research has uncovered a link between transcription dynamics and alternative splicing [41]. It is believed that aberrant splicing can cause disease and a number of studies have tried to understand the mechanisms of alternative splicing [42]. The proposed model can potentially be used to identify transcriptional pausing events, and such results could be usefully combined with inference of splice variation from RNA-Seq datasets from the same system. Also, with the increasing availability of high-throughput sequencing data exploring multiple layered views of the transcription process and its regulation, the convolved modelling approach developed here has the potential to be usefully applied to more complex coupled spatio-temporal datasets.

## Supporting Information

File S1 **Gene lists and clustering results.** The files in this archive include the list of 2623 genes found to exhibit differential pol-II occupancy between 0 and 40 min after E2 treatment and also greater than 1000 bp in length. A BED file with the coordinates of the genes according to the hg19 annotation and a list of 383 genes found to fit the pol-II model well and their cluster indices.
(ZIP)

Text S1 **Supporting text**. This file contains additional details of the mathematical model and results of biological validation via gene ontology analysis and transcription factor binding.
(PDF)
