# Supplementary material for: Inference of RNA Polymerase II Transcription Dynamics from Chromatin Immunoprecipitation Time Course Data
Source: PLoS Comput Biol. 2014 May 15;10(5):e1003598. doi: 10.1371/journal.pcbi.1003598 (PMC4022483; doi:10.1371/journal.pcbi.1003598)
Supplement: Text S1 — Supporting text. This file contains additional details of the mathematical model and results of biological validation via gene ontology analysis and transcription factor binding. (PDF) [file pcbi.1003598.s002.pdf]

Supplementary Information for "Inference of RNA Polymerase II Transcription Dynamics from Chromatin Immunoprecipitation Time Course Data"

Ciira wa Maina, Antti Honkela, Filomena Matarese, Korbinian Grote, Hendrik G. Stunnenberg, George Reid, Neil D. Lawrence, and Magnus Rattray

Priors

The parameters $\Theta = \{\sigma_f, \ell_f, \{\alpha_i, D_i, \ell_i, \sigma_i\}_{i=1}^{I}\}$ are positive and bounded. In the experiments we use the bounds shown in Table S1 with D_1 fixed at zero, $\sigma_f = 1$ and the values σ_i tied to single value. To determine the delay bounds, we assume that the value of D_i is an indicator of how long it takes the 'transcription wave' to reach the corresponding gene segment. That is D_2 is the amount of time it takes to transcribe 20% of the gene, D_3 40% etc. We obtain the length L of the gene from the hg19 annotation and use values of maximum and minimum expected speed (s_{min} and s_{max} respectively) to compute the delay bound. For example

$$D_2^{min} = \frac{0.2L}{s_{max}} \quad \text{and} \quad D_2^{max} = \frac{0.2L}{s_{min}}$$

We use $s_{min} = 50$ bp min^{-1} and $s_{max} = 50$ kbp min^{-1}. These large bounds allow unbiased estimation of transcription speed. (Recent work on individual cells suggests speeds as high as 50kb per minute are possible [1].)

We transform the parameters using a logit transform and work with unconstrained variables. For a parameter $\theta \in \Theta$ with corresponding minimum and maximum bounds θ_{min} and θ_{max} respectively we compute the transfromed variable γ

$$\gamma = \log\left(\frac{\theta - \theta_{min}}{\theta_{max} - \theta}\right). \tag{1}$$

We place a Gaussian prior over the parameters in the transformed domain and draw samples from the posterior using the Hamiltonian Monte Carlo (HMC) algorithm [2]. We have

$$\gamma \sim \mathcal{N}(\gamma|0, \sigma_\gamma). \tag{2}$$

With $, \sigma_\gamma = 2$ we obtain an approximately uniform prior in the untransformed domain yielding an uninformative prior.

To initialise the parameters for gradient optimisation, the length scales ℓ_f and ℓ_i are initilised at random from $\{10, 20, 30, 40, 80\}$, α_i and σ_i are drawn from $\mathcal{U}[0, 1]$ with the value of σ_i multiplied by 100 to avoid local minima that would under-estimate the variance. The delays are inilialised at random with the more realistic speed bounds s_{min}=500 bp per min and s_{max} = 5kb per min when an ensemble of cells is considered. The parameters are then freely optimised with the bounds given in Table S1.

Parameter	Minimum	Maximum
ℓ_f	5 min	320 min
α_i	0	100
D_i	$\frac{0.2(i-1)L}{s_{max}}$ min	$\frac{0.2(i-1)L}{s_{min}}$ min
ℓ_f	5 min	320 min
σ_i	0	100

Table S1: Parameter bounds.

Parameter gradients

To obtain ML estimates of the parameters we maximise the log marginal likelihood. To do this we require the gradients of the covariance function w.r.t the parameters. The gradients w.r.t α_i and σ_i are straight forward. Here we give the expressions for the gradients of $\mathsf{cov}[y_i(t), y_j(t')] = K_{yy}$ w.r.t ℓ_f, ℓ_i and D_i. We have

$$\begin{aligned} \frac{\partial K_{yy}}{\partial \ell_f} &= \alpha_i \alpha_j \frac{\sigma_f^2(\ell_i^2 + \ell_j^2)}{(\ell_f^2 + \ell_i^2 + \ell_j^2)^{\frac{3}{2}}} \exp\left(- \frac{(t' - t + D_i - D_j)^2}{2(\ell_f^2 + \ell_i^2 + \ell_j^2)} \right) \\ &+ \alpha_i \alpha_j \frac{\sigma_f^2 \ell_f}{\sqrt{\ell_f^2 + \ell_i^2 + \ell_j^2}} \exp\left(- \frac{(t' - t + D_i - D_j)^2}{2(\ell_f^2 + \ell_i^2 + \ell_j^2)} \right) \frac{(t' - t + D_i - D_j)^2}{(\ell_f^2 + \ell_i^2 + \ell_j^2)^2} \end{aligned} \tag{3}$$

$$\begin{aligned} \frac{\partial K_{yy}}{\partial \ell_i} &= -\alpha_i \alpha_j \frac{\sigma_f^2 \ell_f \ell_i}{(\ell_f^2 + \ell_i^2 + \ell_j^2)^{\frac{3}{2}}} \exp\left(- \frac{(t' - t + D_i - D_j)^2}{2(\ell_f^2 + \ell_i^2 + \ell_j^2)} \right) \\ &+ \alpha_i \alpha_j \frac{\sigma_f^2 \ell_f}{\sqrt{\ell_f^2 + \ell_i^2 + \ell_j^2}} \exp\left(- \frac{(t' - t + D_i - D_j)^2}{2(\ell_f^2 + \ell_i^2 + \ell_j^2)} \right) \frac{\ell_i (t' - t + D_i - D_j)^2}{(\ell_f^2 + \ell_i^2 + \ell_j^2)^2} \end{aligned} \tag{4}$$

$$\frac{\partial K_{yy}}{\partial D_i} = -\alpha_i \alpha_j \frac{\sigma_f^2 \ell_f}{\sqrt{\ell_f^2 + \ell_i^2 + \ell_j^2}} \exp\left(- \frac{(t' - t + D_i - D_j)^2}{2(\ell_f^2 + \ell_i^2 + \ell_j^2)} \right) \frac{(t' - t + D_i - D_j)}{(\ell_f^2 + \ell_i^2 + \ell_j^2)} \tag{5}$$

To obtain gradient w.r.t the transformed parameters given by equation 1, we employ the chain rule.

$$\begin{aligned} \frac{\partial K_{yy}}{\partial \gamma} &= \frac{\partial K_{yy}}{\partial \theta} \frac{\partial \theta}{\partial \gamma} \\ &= \frac{\partial K_{yy}}{\partial \theta} \frac{\exp(\gamma)(\theta_{max} - \theta_{min})}{(1 + \exp(\gamma))^2} \end{aligned} \tag{6}$$

Canonical Pathway and Gene Ontology Analysis

To determine the biological significance of the 383 genes found to fit the pol-II dynamics model well, we used the Genomatix Pathway System (GePS) to look for enriched canonical pathways and gene ontology categories. Table S2 shows the significant canonical pathways (p-value < 0.05)

and the observed genes. It is interesting to note that the pair of genes *JAK1* and *JAK2* are responsible for a large number of the significant canonical pathways. These genes have previously been suggested as potential drug targets in breast cancer (see for example [3]). The enrichment of the FOXA1 transcriptional network provides further confirmation that our model identifies biologically relevant genes. In recent work, Hurtado *et al.* [4] showed that FOXA1 influences the interaction of ERα and chromatin and therefore influences the response of breast cancer cells to E2. Genes in the FOXA1 canonical network found to fit the pol-II model well include *NRIP1* which is believed to be a direct E2 target that mediates the repression of ERα target genes later in the time course[5, 6]. Table S3 shows the top 20 significant gene ontology terms (p-value < 0.05) for molecular function.

Canonical pathway	Genes
IL-6 signaling pathway(JAK1 JAK2 STAT3)	JAK1, JAK2
IFN gamma signaling pathway	JAK1, JAK2
Proteasome complex	PSME1, PSMA4, PSMB5, PSMA2
IL-3 signaling pathway(JAK1 JAK2 STAT5)	JAK1, JAK2
Stat3 signaling pathway	JAK1, JAK2
FOXA1 transcription factor network	AP1B1, NDUFV3, NRIP1, SHH
PDGFR-alpha signaling pathway	JAK1, PDGFB, SHB
Hypoxia and p53 in the cardiovascular system	FHL2, HIF1A, GADD45A
LIF signaling pathway	JAK1, JAK2
IL-5 signaling pathway	JAK1, JAK2
p53 signaling pathway	TIMP3, GADD45A
IL-10 anti-inflammatory signaling pathway	JAK1, BLVRB
AndrogenReceptor	SPDEF, FHL2, STUB1 NCOR2, NRIP1
Integrin signaling pathway	CSK, ACTN1, NOLC1
Erythropoietin mediated neuroprotection through NF-KB	HIF1A, JAK2
PDGFR-beta signaling pathway	ACTR2, HCK, CSK, PDGFB, CTTN, JAK2
Mechanisms of transcriptional repression by dna methylation	RBBP7, MBD1
Hypoxia-inducible factor in the cardivascular system	HIF1A, LDHA

Table S2: Significant canonical pathways (p-value < 0.05) for the 383 genes found to fit the pol-II dynamics model well.

Table S4 shows the significant canonical pathways (p-value < 0.01) and the observed genes in each of the 12 promoter profile clusters. We also perform a gene ontology analysis of the 12 promoter profile clusters using the DAVID tool from the NIH [7, 8]. The enriched gene ontology categories (p-value < 0.05) are shown in Table S5, (for molecular function), Table S6 (for biological processes) and Table S7 (for cellular components).

Molecular function
Structural constituent of ribosome
RNA binding
Methyl-CpG binding
Protein binding
Structural molecule activity
Nucleic acid binding
rRNA binding
Non-membrane spanning protein tyrosine kinase activity
Ribosomal small subunit binding
Pseudouridine synthase activity
S100 alpha binding
Growth hormone receptor binding
Isomerase activity
Glucocorticoid receptor binding
Translation factor activity, nucleic acid binding
NF-kappaB binding
Threonine-type peptidase activity
Threonine-type endopeptidase activity
Intramolecular transferase activity

Table S3: Top 20 significant gene ontology terms (p-value < 0.05) for the 383 genes found to fit the pol-II dynamics model well.

Cluster	Canonical pathway	Genes
1 (37)	PDGFR-beta signaling pathway	PDGFB, ACTR2, HCK
2 (47)	-	-
3 (18)	-	-
4 (29)	Nuclear receptors coordinate the activities of chromatin remodeling complexes and coactivators to facilitate initiation of transcription in carcinoma cells	NCOR2, TAF5
5 (27)	-	-
6 (40)	-	-
7 (24)	Proteasome complex<br>Antigen processing and presentation	PSMB5, PSME1<br>PSMB5
8 (47)	-	-
9 (26)	-	-
10 (38)	IFN gamma signaling pathway<br>IL-6 signaling pathway<br>IL-3 signaling pathway<br>Stat3 signaling pathway<br>LIF signaling pathway<br>IL-5 signaling pathway<br>PDGFR-alpha signaling pathway<br>IL27-mediated signaling events<br>Role of ErbB2 in signal transduction and oncology<br>IL6-mediated signaling events<br>JAK_STAT_MolecularVariation_2	JAK2, JAK1<br>JAK2, JAK1<br>JAK2, JAK1<br>JAK2, JAK1<br>JAK2, JAK1<br>JAK2, JAK1<br>SHB, JAK1<br>JAK2, JAK1<br>JAK2, JAK1<br>JAK2, JAK1<br>JAK2, JAK1
11 (13)	-	-
12 (37)	-	-

Table S4: Pathway analysis of clusters from inferred promoter activity profiles. The number in parentheses in column 1 is the cluster size.

Cluster	GO ID	GO TERM
1 (37)	GO:0008092	Cytoskeletal protein binding
	GO:0003779	Actin binding
	GO:0005085	Guanyl-nucleotide exchange factor activity
2 (47)	GO:0003723	RNA binding
3 (18)	-	-
4 (29)	GO:0003723	RNA binding
	GO:0030528	transcription regulator activity
	GO:0003677	DNA binding
	GO:0003700	Transcription factor activity
5 (27)	-	-
6 (40)	GO:0003735	Structural constituent of ribosome
7 (24)	GO:0003735	Structural constituent of ribosome
	GO:0005198	Structural molecule activity
	GO:0003723	RNA binding
8 (47)	-	-
9 (26)	GO:0043021	Ribonucleoprotein binding
10 (38)	GO:0005131	Growth hormone receptor binding
	GO:0051427	Hormone receptor binding
	GO:0032553	Ribonucleotide binding
	GO:0032555	Purine ribonucleotide binding
	GO:0017076	Purine nucleotide binding
	GO:0005525	GTP binding
	GO:0019001	Guanyl nucleotide binding
	GO:0032561	Guanyl ribonucleotide binding
	GO:0004713	Protein tyrosine kinase activity
11 (13)	-	-
12 (37)	GO:0003735	Structural constituent of ribosome
	GO:0005198	Structural molecule activity
	GO:0003723	RNA binding

Table S5: Enriched gene ontology categories for molecular function (p-value < 0.05) of clusters from inferred promoter activity profiles. The number in parentheses in column 1 is the cluster size.

Cluster	GO ID	GO TERM
1 (37)	GO:0030036	Actin cytoskeleton organization
	GO:0030029	Actin filament-based process
	GO:0007010	Cytoskeleton organization
	GO:0007517	Muscle organ development
	GO:0001503	Ossification
	GO:0001501	Skeletal system development
	GO:0060348	Bone development
	GO:0060537	Muscle tissue development
	GO:0051496	Positive regulation of stress fiber formation
	GO:0007167	Enzyme linked receptor protein signaling pathway
	GO:0045935	Positive regulation of nucleobase, nucleoside, nucleotide and nucleic acid metabolic process
	GO:0032233	Positive regulation of actin filament bundle formation
	GO:0051173	Positive regulation of nitrogen compound metabolic process
	GO:0010557	Positive regulation of macromolecule biosynthetic process
	GO:0031328	Positive regulation of cellular biosynthetic process
	GO:0009891	Positive regulation of biosynthetic process
	GO:0051492	Regulation of stress fiber formation
	GO:0048008	Platelet-derived growth factor receptor signaling pathway
	GO:0032231	Regulation of actin filament bundle formation
	GO:0055010	Ventricular cardiac muscle morphogenesis
	GO:0055008	Cardiac muscle tissue morphogenesis
	GO:0060415	Muscle tissue morphogenesis
2 (47)	GO:0051272	Positive regulation of cell motion
	GO:0043085	Positive regulation of catalytic activity
	GO:0044093	Positive regulation of molecular function
3 (18)	GO:0006364	rRNA processing
	GO:0016072	rRNA metabolic process
4 (29)	GO:0010558	Negative regulation of macromolecule biosynthetic process
	GO:0031327	Negative regulation of cellular biosynthetic process
	GO:0006350	Transcription
	GO:0009890	Negative regulation of biosynthetic process
	GO:0010605	Negative regulation of macromolecule metabolic process
	GO:0016481	Negative regulation of transcription
	GO:0010629	Negative regulation of gene expression
	GO:0045934	Negative regulation of nucleobase, nucleoside, nucleotide and nucleic acid metabolic process
	GO:0051172	Negative regulation of nitrogen compound metabolic process
5 (27)	-	-
6 (40)	GO:0048147	Negative regulation of fibroblast proliferation
	GO:0022613	Ribonucleoprotein complex biogenesis
7 (24)	GO:0019941	Modification-dependent protein catabolic process
	GO:0043632	Modification-dependent macromolecule catabolic process
	GO:0051603	Proteolysis involved in cellular protein catabolic process
	GO:0044257	Cellular protein catabolic process

	GO:0030163	Protein catabolic process
	GO:0006412	Translation
	GO:0043161	Proteasomal ubiquitin-dependent protein catabolic process
	GO:0010498	Proteasomal protein catabolic process
	GO:0044265	Cellular macromolecule catabolic process
	GO:0009057	Macromolecule catabolic process
	GO:0006508	Proteolysis
	GO:0006511	Ubiquitin-dependent protein catabolic process
8 (47)	GO:0042273	Ribosomal large subunit biogenesis
	GO:0006396	RNA processing
	GO:0006400	tRNA modification
9 (26)	GO:0043086	Negative regulation of catalytic activity
10 (38)	GO:0007242	Intracellular signaling cascade
	GO:0015031	Protein transport
	GO:0045184	Establishment of protein localization
	GO:0008104	Protein localization
	GO:0001525	Angiogenesis
	GO:0010876	Lipid localization
11 (13)	-	-
12 (37)	GO:0006412	Translation
	GO:0006414	Translational elongation
	GO:0051168	Nuclear export
	GO:0042274	Ribosomal small subunit biogenesis
	GO:0000278	Mitotic cell cycle
	GO:0006974	Response to DNA damage stimulus
	GO:0006913	Nucleocytoplasmic transport
	GO:0051169	Nuclear transport
	GO:0022613	Ribonucleoprotein complex biogenesis

Table S6: Enriched gene ontology categories for biological processes (p-value < 0.05) of clusters from inferred promoter activity profiles. The number in parentheses in column 1 is the cluster size.

Cluster	GO ID	GO TERM
1 (37)	GO:0015629	Actin cytoskeleton
	GO:0005856	Cytoskeleton
	GO:0043228	Non-membrane-bounded organelle
	GO:0043232	Intracellular non-membrane-bounded organelle
	GO:0030017	Sarcomere
	GO:0030016	Myofibril
	GO:0044449	Contractile fiber part
	GO:0043292	Contractile fiber
	GO:0001725	Stress fiber
2 (47)	-	-
3 (18)	-	-
4 (29)	GO:0016604	Nuclear body
	GO:0005654	Nucleoplasm
	GO:0030529	Ribonucleoprotein complex
	GO:0044451	Nucleoplasm part
	GO:0031981	Nuclear lumen
	GO:0022625	Cytosolic large ribosomal subunit
5 (27)	GO:0030529	Ribonucleoprotein complex
	GO:0005732	Small nucleolar ribonucleoprotein complex
	GO:0043232	Intracellular non-membrane-bounded organelle
	GO:0043228	Non-membrane-bounded organelle
6 (40)	GO:0044429	Mitochondrial part
	GO:0070013	Intracellular organelle lumen
	GO:0043233	Organelle lumen
	GO:0031974	Membrane-enclosed lumen
	GO:0005743	Mitochondrial inner membrane
	GO:0019866	Organelle inner membrane
	GO:0044455	Mitochondrial membrane part
	GO:0033279	Ribosomal subunit
	GO:0031966	Mitochondrial membrane
	GO:0005739	Mitochondrion
	GO:0005740	Mitochondrial envelope
	GO:0005840	Ribosome
7 (24)	GO:0005840	Ribosome
	GO:0033279	Ribosomal subunit
	GO:0030529	Ribonucleoprotein complex
	GO:0000313	Organellar ribosome
	GO:0005761	Mitochondrial ribosome
8 (47)	GO:0031981	Nuclear lumen
	GO:0005730	Nucleolus
	GO:0070013	Intracellular organelle lumen
	GO:0043233	Organelle lumen
	GO:0031974	Membrane-enclosed lumen
	GO:0030529	Ribonucleoprotein complex
9 (26)	GO:0031981	Nuclear lumen

10 (38)	GO:0009898	Internal side of plasma membrane
	GO:0044459	Plasma membrane part
11 (13)	GO:0022625	Cytosolic large ribosomal subunit
	GO:0015934	Large ribosomal subunit
	GO:0022626	Cytosolic ribosome
12 (37)	GO:0005840	Ribosome
	GO:0033279	Ribosomal subunit
	GO:0030529	Ribonucleoprotein complex
	GO:0043232	Intracellular non-membrane-bounded organelle
	GO:0043228	Non-membrane-bounded organelle
	GO:0044445	Cytosolic part
	GO:0005761	Mitochondrial ribosome
	GO:0000313	Organellar ribosome
	GO:0015935	Small ribosomal subunit
	GO:0015934	Large ribosomal subunit
	GO:0031980	Mitochondrial lumen
	GO:0005759	Mitochondrial matrix
	GO:0022626	Cytosolic ribosome
	GO:0005829	Cytosol
	GO:0070013	Intracellular organelle lumen
	GO:0043233	Organelle lumen
	GO:0031974	Membrane-enclosed lumen
	GO:0005739	Mitochondrion
	GO:0000315	Organellar large ribosomal subunit
	GO:0005762	Mitochondrial large ribosomal subunit

Table S7: Enriched gene ontology categories for cellular components (p-value < 0.05) of clusters from inferred promoter activity profiles. The number in parentheses in column 1 is the cluster size.

Clustering the raw ChIP-Seq reads

Pol-II occupancy in the proximal promoter region -250 bp to +750 bp relative to the transcription start site (TSS) was computed in RPM for the 383 genes and the time series grouped into 12 clusters. The clusters are shown in Figure S1. Table S8 shows the significant canonical pathways (p-value < 0.01) and the observed genes in each of the 12 clusters. We find that in this case *JAK1* and *JAK2* appear in different clusters which have diffent temporal profiles. This may be due to the noisy nature of the data and the inclusion of paused pol-II in the proximal region time series. Our model which has the potential to uncover the signal due to pol-II that is engaged in transcription could be useful in uncovering relationships which may be missed if we only consider the raw ChIP-seq reads.

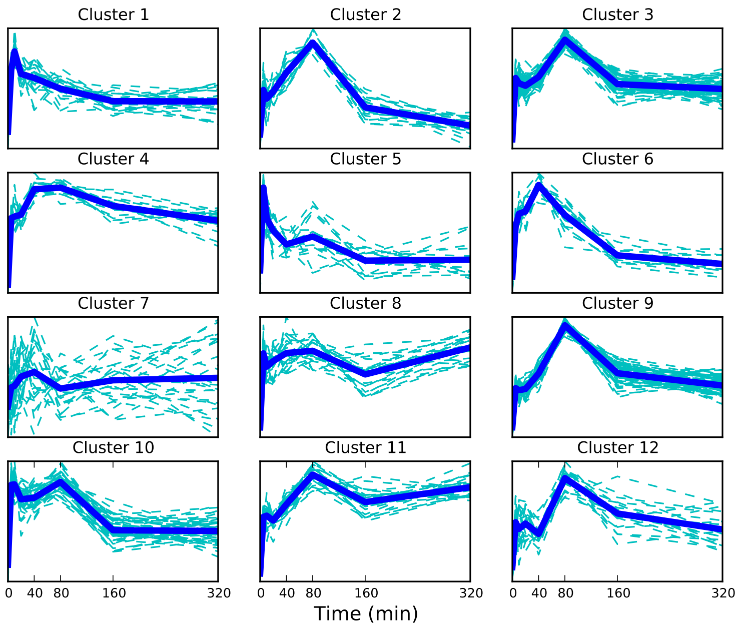

Figure S1: Clusters of promoter activity profiles derived directly from the raw ChIP-seq reads. The mean profile in each cluster is shown by the bold line.

Cluster	Canonical pathway	Genes
1 (24)	Transcriptional activation of dbpB from mRNA	PDGFB
2 (23)	-	-
3 (75)	Hypoxia and p53 in the cardiovascular system	GADD45A, HIF1A
4 (18)	Generation of amyloid b-peptide by ps1	ATP5G3
5 (16)	PDGFR-alpha signaling pathway	SHB, JAK1
	IFN gamma signaling pathway	JAK1
	IL-6 signaling pathway	JAK1
	IL-10 signaling pathway	JAK1
6 (15)	-	-
7 (24)	-	-
8 (24)	-	-
9 (67)	Proteasome complex	PSME1, PSMB5, PSMA4
10 (49)	TPO signaling pathway	JAK2
11 (29)	Glypican 3 network	SHH
	Sonic hedgehog receptor ptc1 regulates cell cycle	SHH
12 (19)	Hypoxia-inducible factor in the cardivascular system	LDHA
	Fibrinolysis pathway	ATP2A2

Table S8: Pathway analysis of clusters from raw ChIP-seq reads in the proximal promoter region -250bp to +750bp from the TSS. The number in parentheses in column 1 is the cluster size.

Transcription Factor Binding

Motifs

Tullai *et al.* [9] investigated genes that are co-regulated by shared transcription factor binding sites (TFBS). In particular, they found certain TFBS were enriched in the promoters of early response

genes. We therefore investigated whether the promoters of genes in the different promoter profile clusters are enriched for different TFs. We use Pscan [10] to look for enriched TF motifs among those available in JASPAR [11]. The proximal promoter region -450 bp to +50 bp relative to the TSS of the genes in each cluster was analyzed. Table S9 shows signifiantly enriched TFBS in each cluster (p-value < 0.05). Shown are TFs whose binding sites are over-represented in at least 5 clusters. The estrogen response element (ERE) is enriched in five clusters (1, 2, 5, 6 and 10), indicating that our modelling identifies estrogen responsive regions. The clusters containing an ERE have mean promoter activity profiles with distinct early peaks followed by decrease in activity which suggests transient activity. Additionally, clusters 1, 2 and 10 have relatively early peaks.

TF	Cluster											
	1	2	3	4	5	6	7	8	9	10	11	12
GABPA	✓	✓	✓	✓	✓	-	✓	✓	✓	-	✓	✓
Zfx	✓	✓	-	✓	✓	-	-	✓	✓	✓	✓	✓
Klf4	✓	✓	-	✓	-	-	✓	✓	✓	✓	-	✓
ELK1	✓	-	-	✓	✓	-	✓	✓	✓	-	✓	✓
HIF1A::ARNT	✓	✓	-	✓	✓	✓	✓	-	-	✓	✓	-
ELK4	✓	-	✓	✓	✓	-	✓	✓	✓	-	-	✓
SP1	✓	✓	-	✓	-	-	-	✓	✓	✓	-	✓
TFAP2A	✓	✓	-	✓	-	✓	-	✓	-	✓	-	-
Mycn	✓	-	-	✓	✓	-	-	✓	-	✓	✓	-
Myc	✓	-	-	✓	✓	-	-	✓	-	✓	✓	-
Pax5	✓	✓	-	✓	-	-	-	-	-	✓	-	✓
**ER**α	✓	✓	-	-	✓	✓	-	-	-	✓	-	-
Arnt::Ahr	-	✓	-	✓	-	-	✓	✓	-	✓	-	-

Table S9: Significantly over-represented (p-value < 0.05) transcription factor binding sites in the promoter profile clusters. We use Pscan to look for enriched TF motifs among those available in JASPAR. The proximal promoter region -450 bp to +50 bp relative to the TSS of the genes in each cluster was analyzed.

Next we investigated the EREs in the genes belonging to the 5 clusters enriched for the ERE motif. For each promoter sequence, the best sequence match to the ERE position frequency matrix (PFM) in JASPAR (MA0112.2) was determined. We keep those sequences with a matrix score greater than the mean score for matches found in the promoter sequences over the whole genome (For the ERE PFM this value is 0.73 when we consider the region -450 bp to +50 bp relative to the TSS). We used these sequences to determine the consensus ERE motif in this group of genes. To determine the consensus sequence, we report a single nucleotide for a given position if the nucleotide has a frequency greater than 50% and a frequency twice as large as the next nucleotide. We obtain a consensus sequence of 5'-GGnCACCCTGnCC-3' (where n is any nucleotide) and an average matrix score of 0.77. The sequence is visualised in Figure S2 (A). The sequence of the ERE is known and given as 5'-GGTCAnnnTGACC-3' [12, 13]. The sequence corresponding to the PFM MA0112.2 is visualised in Figure S2 (B). We see that the ERE motif we obtain agrees well with the known motif.

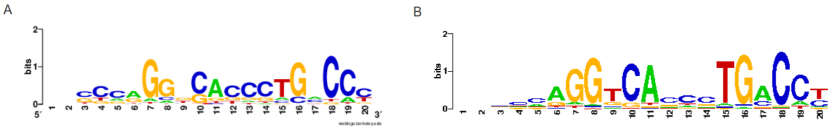

Figure S2: Consensus sequence of regions matching the ERE motif in the promoter profile clusters enriched for the ERE motif (A) and the Estrogen Response Element (B).

Table S10 shows the EREs in each of the 5 clusters visualised using WebLogo. We also show the consensus sequence and the average matrix score. To determine the consensus sequence, we report a single nucleotide for a given position if the nucleotide has a frequency greater than 50% and a frequency twice as large as the next nucleotide. We see that there is some diversity in the motifs correspoding to different clusters but the consensus sequences agree with the known motif. Differences appear at at most 3 positions with the consensus sequence for cluster 10 differing at only two positions. We see that for the half site 'TGACC' the 'A' does not appear in the consensus sequence in all the clusters.

Transcription factor binding

Determining the TFBS motifs enriched in each cluster provides a way to determine the influence of TFs on transcription. As a complementary approach, we also investigated the TF peaks in regions ranging from 1 to 100 kb around the gene transcription start site for all genes in each cluster using ChIP-seq data for a number of TFs measured under similar experimental conditions (i.e. MCF-7 breast cancer cells treated with E2) in the cistrome database (`http://cistrome.org`).

Tables S11 to S14 show the number of genes with TF binding peaks for regions around the TSS ranging from 1 to 100 kb for each cluster for 7 TFs namely ERα [14], FoxA1 [15], c-Fos [16], c-Jun [16], c-MYC [17], SRC-3 [18], TRIM24 [19]. In the tables, statistically significant (p-value < 0.05) proportions are indicated in red (larger than expected) and green (lower than expected) with associated p-values in parentheses. These p-values are obtained empirically by drawing 1e6 samples from a hypergeometric distribution.

We investigated the overlap of the binding sites for ERα and FOXA1 both in the 151 genes belonging to the rapid response genes in clusters 1, 2, 4, and 10 and genome-wide using the peaks obtained from [14] (ERα) and [15] (FOXA1) and reported in the cistrome database. We investigated regions around the TSS ranging from 2 to 100 kb. Tables S15-S18 show the number of ERα and FOXA1 peaks and the overlap. The statistical significance is determined by comparing the overlap in random gene lists of the same size.

Cluster	ERE Motif	Consensus sequence	Average Matrix Score
1	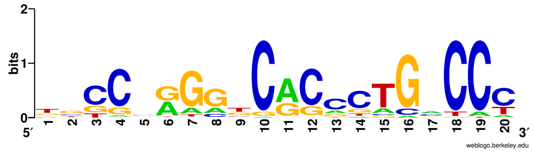	GnnCACCCTGnCCC	0.772
2	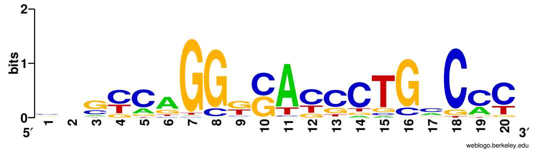	GGnnACCCTGnCCn	0.77
5	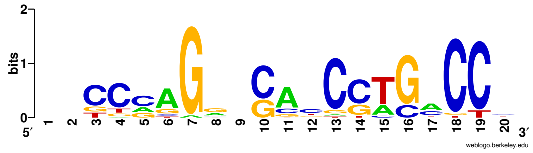	GGnnAnCCTGnCCn	0.761
6	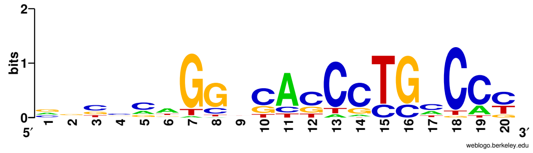	GGnnACCnTGnCCn	0.762
10	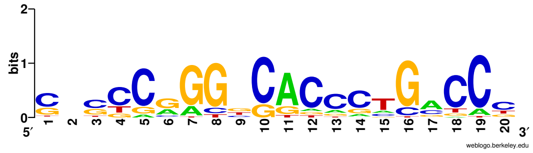	GGnCACCCTGnCCn	0.765

Table S10: Analysis of the ERE in promoter regions of gene clusters obtained from inferred promoter activity profiles. The EREs in each of the 5 clusters are visualised using WebLogo (http://weblogo.berkeley.edu/). The consensus sequence is shown from postion 7 which corresponds to the known ERE motif. The average matrix score is computed using the sequence matrix scores from Pscan.

Cluster	TFs						
	ERα	FOXA1	c-FOS	c-JUN	MYC	SRC-3	TRIM24
1 (37)	5	4	2	3	1	7	9
2 (47)	9 (*)	3	2	2	4	12 (*)	10
3 (18)	3	2	2	1	3 (*)	3	2
4 (29)	4	2	1	0 (***)	0 (***)	3	5
5 (27)	3	0 (***)	0 (***)	5 (*)	4 (*)	7	5
6 (40)	5	3	3	0 (***)	3	8	2
7 (24)	1	2	0 (***)	3	1	6	7
8 (47)	3	2	1	3	4	6	14 (*)
9 (26)	2	2	4	5 (**)	1	5	6
10 (38)	9 (*)	2	1	0 (***)	0 (***)	3	9
11 (13)	0 (***)	0 (***)	3	1	1	1	1
12 (37)	5	0 (***)	2	5 (*)	2	11 (**)	7

Table S11: Analysis of transcription factor binding in 1kbp regions of genes in gene clusters obtained from inferred promoter activity profiles. The number in parentheses in the first column is the cluster size. For each TF, we show the number of genes with peaks. Statistically significant proportions (p-value < 0.05) are indicated in red (larger than expected). For p-values less than 0.01, the associated p-values are indicated in parentheses according to the following scale (***: $p < 0.0001$,**: $p < 0.001$,*:$p < 0.01$).

Cluster	TFs						
	ERα	FOXA1	c-FOS	c-JUN	MYC	SRC-3	TRIM24
1 (37)	8	4	3	3	1	8	10
2 (47)	10	3	3	2	5 (*)	14 (**)	11
3 (18)	3	2	2	1	3	3	3
4 (29)	4	2	1	0 (***)	0 (***)	3	9
5 (27)	4	0 (***)	1	5 (*)	6 (***)	8	6
6 (40)	9	5	5	0 (***)	3	11 (*)	3
7 (24)	2	3	0 (***)	3	1	7	10 (*)
8 (47)	5	2	1	4	4	9	19 (**)
9 (26)	3	2	6 (*)	6 (**)	1	7	7
10 (38)	11 (*)	3	2	0 (***)	1	5	10
11 (13)	1	0 (***)	3	1	1	1	3
12 (37)	6	0 (***)	2	5 (*)	2	11 (*)	8

Table S12: Analysis of transcription factor binding in 2kbp regions.

Cluster	TFs						
	ERα	FOXA1	c-FOS	c-JUN	MYC	SRC-3	TRIM24
1 (37)	20 (*)	9	8	4	1	18	22
2 (47)	24 (*)	13	12	6	7 (*)	30 (***)	28
3 (18)	4	4	4	2	5 (*)	8	7
4 (29)	11	6	4	2	1	12	18
5 (27)	9	2	3	6 (*)	8 (***)	11	14
6 (40)	22 (**)	8	6	4	3	18	24
7 (24)	7	4	2	4	2	13	16
8 (47)	21	6	7	10 (*)	7 (*)	28 (***)	34 (**)
9 (26)	10	4	8	9 (***)	1	8	20 (*)
10 (38)	26 (***)	11	9	0 (***)	1	21 (*)	24
11 (13)	4	0 (***)	5	2	1	4	8
12 (37)	12	2	7	10 (**)	4	20 (*)	23

Table S13: Analysis of transcription factor binding in 20kbp regions.

Cluster	TFs						
	ERα	FOXA1	c-FOS	c-JUN	MYC	SRC-3	TRIM24
1 (37)	29	20	26 (***)	12	4	32 (*)	36
2 (47)	41 (*)	26	23	11	12 (*)	43 (**)	43
3 (18)	17	7	10	6	6	14	16
4 (29)	29 (***)	17	15	10	5	25	28
5 (27)	21	8	11	12 (*)	11 (**)	19	24
6 (40)	36 (*)	15	19	11	6	35 (*)	38
7 (24)	15	11	8	9	5	18	22
8 (47)	42 (**)	20	22	15	9	41 (*)	45
9 (26)	23	15	16 (*)	12 (**)	5	22	24
10 (38)	34 (*)	27 (**)	20	5	4	34 (*)	36
11 (13)	9	4	8	4	2	10	13
12 (37)	31	11	19	14 (*)	5	28	35

Table S14: Analysis of transcription factor binding in 100kbp regions.

Genes	# of ERα peaks	# of FOXA1 peaks	ERα and FOXA1 overlap
Clusters 1, 2, 4, and 10 (151)	28 (12)	11 (6)	7 (0.042)
All genes (∼ 20,000)	1596	758	130

Table S15: Overlap of ERα and FOXA1 binding in a 1 kb region around the TSS. The numbers in parentheses in the first column are the number of genes. In each TF peak column, we show the expected number of peaks in a set of random random genes of the same size in parentheses. In the overlap column the associated p-value is shown in parentheses.

Genes	# of ERα peaks	# of FOXA1 peaks	ERα and FOXA1 overlap
Clusters 1, 2, 4, and 10 (151)	36 (17)	13 (7)	8 (0.038)
All genes (∼ 20,000)	2220	929	177

Table S16: Overlap of ERα and FOXA1 binding in a 2 kb region around the TSS.

Genes	# of ERα peaks	# of FOXA1 peaks	ERα and FOXA1 overlap
Clusters 1, 2, 4, and 10 (151)	125 (63)	44 (26)	19 (0.045)
All genes ($\sim$ 20,000)	7229	2991	626

Table S17: Overlap of ERα and FOXA1 binding in a 20 kb region around the TSS.

Genes	# of ERα peaks	# of FOXA1 peaks	ERα and FOXA1 overlap
Clusters 1, 2, 4, and 10 (151)	488 (254)	171 (100)	66 (0.006)
All genes ($\sim$ 20,000)	17942	7927	1691

Table S18: Overlap of ERα and FOXA1 binding in a 100 kb region around the TSS.

References

[1] Maiuri P, Knezevich A, De Marco A, Mazza D, Kula A, et al. (2011) Fast transcription rates of RNA polymerase II in human cells. EMBO Rep .

[2] Neal RM (2011) MCMC using Hamiltonian dynamics. In: S Brooks, A Gelman, G Jones and X-L Meng, editor, Handbook of Markov Chain Monte Carlo, Chapman and Hall/CRC.

[3] The Cancer Genome Atlas Network (2012) Comprehensive molecular portraits of human breast tumours. Nature 490: 61–70.

[4] Hurtado A, Holmes KA, Ross-Innes CS, Schmidt D, Carroll JS (2011) FOXA1 is a key determinant of estrogen receptor function and endocrine response. Nature Genetics 43: 27–33.

[5] Carroll JS, Meyer CA, Song J, Li W, Geistlinger TR, et al. (2006) Genome-wide analysis of estrogen receptor binding sites. Nature Genetics 38: 1289–1297.

[6] Jagannathan V, Robinson-Rechavi M (2011) Meta-analysis of estrogen response in MCF-7 distinguishes early target genes involved in signaling and cell proliferation from later target genes involved in cell cycle and DNA repair. BMC Syst Biol 5: 138.

[7] Huang DW, Sherman BT, Lempicki RA (2008) Systematic and integrative analysis of large gene lists using DAVID bioinformatics resources. Nat Protocols 4: 44–57.

[8] Huang DW, Sherman BT, Lempicki RA (2009) Bioinformatics enrichment tools: paths toward the comprehensive functional analysis of large gene lists. Nucleic Acids Research 37: 1–13.

[9] Tullai JW, Schaffer ME, Mullenbrock S, Sholder G, Kasif S, et al. (2007) Immediate-early and delayed primary response genes are distinct in function and genomic architecture. J Biol Chem 282: 23981-95.

[10] Zambelli F, Pesole G, Pavesi G (2009) Pscan: finding over-represented transcription factor binding site motifs in sequences from co-regulated or co-expressed genes. Nucleic Acids Research 37: 247-252.

[11] Sandelin A, Alkema W, Engström P, Wasserman WW, Lenhard B (2004) JASPAR: an openaccess database for eukaryotic transcription factor binding profiles. Nucleic Acids Research 32: D91–D94.

[12] Klinge CM (2001) Estrogen receptor interaction with estrogen response elements. Nucleic Acids Research 29: 2905-2919.

[13] Welboren WJ, Stunnenberg HG, Sweep FCGJ, Span PN (2007) Identifying estrogen receptor target genes. Molecular oncology 1: 138–143.

[14] Welboren WJ, van Driel MA, Janssen-Megens EM, van Heeringen SJ, Sweep FCGJ, et al. (2009) ChIP-Seq of ERα and RNA polymerase II defines genes differentially responding to ligands. The EMBO Journal 28: 1418–1428.

[15] Lupien M, Eeckhoute J, Meyer CA, Wang Q, Zhang Y, et al. (2008) FoxA1 translates epigenetic signatures into enhancer-driven lineage-specific transcription. Cell 132: 958–970.

[16] Joseph R, Orlov YL, Huss M, Sun W, Kong SLL, et al. (2010) Integrative model of genomic factors for determining binding site selection by estrogen receptor α. Molecular systems biology 6: 456.

[17] Hua S, Kittler R, White KP (2009) Genomic antagonism between retinoic acid and estrogen signaling in breast cancer. Cell 137: 1259–1271.

[18] Lanz RB, Bulynko Y, Malovannaya A, Labhart P, Wang L, et al. (2010) Global Characterization of Transcriptional Impact of the SRC-3 Coregulator. Molecular Endocrinology 24: 859-872.

[19] Tsai WW, Wang Z, Yiu TT, Akdemir KC, Xia W, et al. (2010) TRIM24 links a non-canonical histone signature to breast cancer. Nature 468: 927–932.
